# Design, synthesis and biological assessment of new 1-benzyl-4-((4-oxoquinazolin-3(4*H*)-yl)methyl) pyridin-1-ium derivatives (BOPs) as potential dual inhibitors of acetylcholinesterase and butyrylcholinesterase

**DOI:** 10.1016/j.heliyon.2021.e06683

**Published:** 2021-04-08

**Authors:** Samaneh Zarei, Mohammad Shafiei, Maryam Firouzi, Loghman Firoozpour, Kouros Divsalar, Ali Asadipour, Tahmineh Akbarzadeh, Alireza Foroumadi

**Affiliations:** a Department of Medicinal Chemistry, Faculty of Pharmacy and Drug Design & Development Research Center, The Institute of Pharmaceutical Sciences (TIPS), Tehran University of Medical Sciences, Tehran, Iran; b Department of Medicinal Chemistry, Faculty of Pharmacy, Birjand University of Medical Sciences, Birjand, Iran; c Neuroscience Research Center, Institute of Neuropharmacology, Kerman University of Medical Sciences, Kerman, Iran; d Pharmaceutical Sciences and Cosmetic Products Research Center, Kerman University of Medical Sciences, Kerman, Iran

**Keywords:** Alzheimer's disease, Chloinestrases, Oxoquinazolin, Pyridinuim salts, In-vitro assay

## Abstract

Alzheimer's disease (AD), is among the most growing neurodegenerative diseases, which is mainly caused by the acetylcholine neurotransmitter loss in the hippocampus and cortex. Emerging of the dual Acetylcholinesterase (AChE)/Butyrylcholinesterase (BuChE) inhibitors has increased for treating Alzheimer disease. In this study, we would like to report the design and synthesis of a new sequence of 1-benzyl-4-((4-oxoquinazolin-3(4*H*)-yl)methyl) pyridin-1-ium derivatives (BOPs) assessed as BuChE and AChE inhibitors. Ellman's approach was used for the evaluation of AChE and BuChE inhibitory activities. Moreover, docking research was conducted to predict the action mechanism. Among all synthesized compounds, 1-(3-bromobenzyl)-3-((4-oxoquinazolin-3(4*H*)-yl)methyl) pyridin-1-ium bromide (BOP-1) was found to be the most active compound with dual activity for inhibition of AChE (IC_50_ = 5.90 ± 0.07μM), and BuChE (IC_50_ = 6.76 ± 0.04μM) and 1-(4-chlorobenzyl)-3-((6,7-dimethoxy-4-oxoquinazolin-3(4*H*)-yl)methyl) pyridin-1-ium chloride (BOP-8) showed the highest AChE inhibitory activity (IC_50_s = 1.11 ± 0.09 μM). The synthesized compounds BOP-1 and BOP-8 could be proposed as valuable lead compounds for further drug discovery development against AD.

## 1 Introduction

Alzheimer's disease (AD) is a progressive neurodegenerative problem, chiefly prevalent in elder people [1]. The number of people suffering from Alzheimer is increasing significantly. Based on to the World Alzheimer Report (2019), about 50 million people were living with AD in 2019 worldwide, and it is estimated that this number will have risen to 152 million by 2050 [2]. Although the disease's molecular source is not still thoroughly perceived, novel therapeutic approaches to alleviate the pathophysiological symptoms of the disease have achieved great advancements in this field [3]. Among diverse hypotheses proposed for the mechanism of AD, cholinergic hypothesis plays a determining role in leading the scientists to discover viable strategies to overcome the disease [4,5,6].

**Figure 1:**
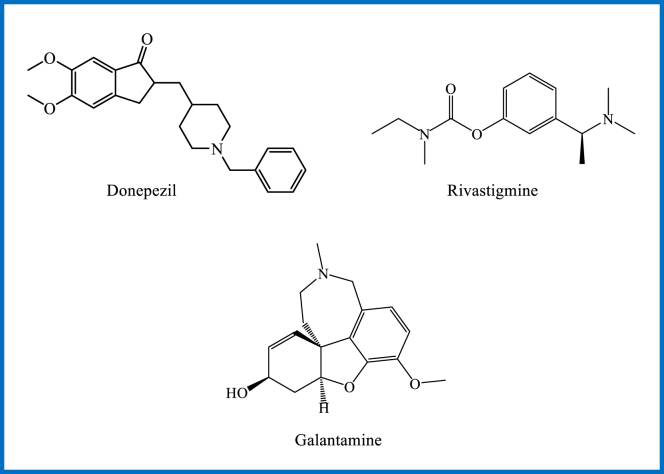
Some of the FDA-approved drugs for treatment of AD.

Based on this theory, a dramatic decline in the acetylcholine neurotransmitter levels in the hippocampus and cortex is accountable for memory loss, learning impairments and cognitive dysfunction [6]. Butyrylcholinesterase (BuChE) and acetylcholineesterase (AChE) are the enzymes accountable for catalyzing the hydrolyzing acetylcholine and consequently generating the clinical manifestations of AD. Therefore, compounds with dual inhibitory effects on AChE and BuChE can be promising candidates for the treatment of AD [7,8]. Galantamine, donepezil and rivastigmine are three widely-available FDA-approved drugs for AD (Figure 1) [9]. Among these drugs, Rivastigmine reveals dual inhibition against BuChE and AChE [10]. Donepezil, a piperidine-based reversible acetylcholinesterase inhibitor, is permitted for the mild-to-moderate AD treatment. In comparison to other AChEIs, donepezil reveals higher cognitive enhancement and more desirable Pharmacokinetic, pharmacodynamic and safety profile [11].

Thus, by designing and synthesizing novel multi-target chemicals with the aim of lowering the adverse effects and heightening the efficacy, management of AD will notably get easier [12].

**Figure 2:**
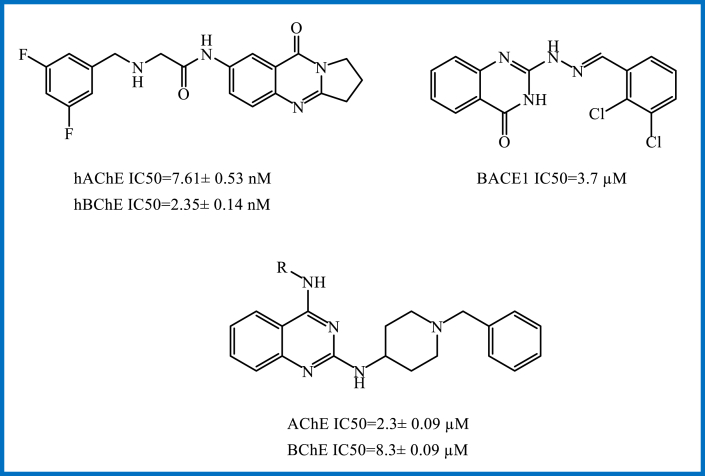
Structures of some quinazoline/quinazolinone based anti-AD agents.

In recent years, the quinazoline/quinazolinone ring scaffold has increasingly gained interest as a privileged structure in a variety of marketed drugs and broad varieties of biologically active compounds, such as anti-microbial, anti-cancer, neuroprotective, and also anti-AD agents, exhibiting inhibition of Aβ aggregation, butyrylcholinesterase (BuChE), and dual acetylcholinesterase (AChE), and have essential scavenging effects (Figure 2) [13,14,15,16,17,18].

Benzyl pyridinium structure has also shown anti-ChE activity. For example, the docking studies, reveals the anti-ChE activity of this moiety through interactions with amino acid residues in the PAS and CAS of AChE and its effectiveness is reported in several SAR studies [19,20,21].

**Figure 3:**
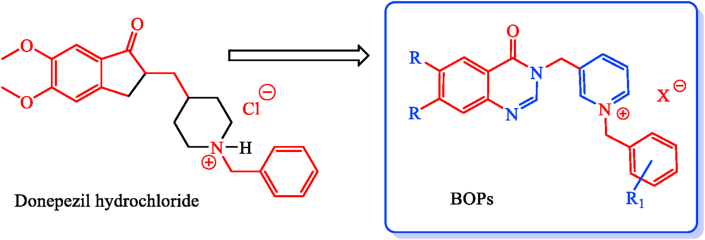
Design strategy for the synthesis of targeted compounds.

In light of the new investigations and following the previous findings, some new 1-benzyl-4-((4-oxoquinazolin-3(4*H*)-yl)methyl) pyridin-1-ium derivatives (BOPs) were designed and synthesized. This modification has been made on donepezil by a scaffold replacement in pursuit of finding potential multi-targeted compounds (Figure 3). Thus, the synthesized compounds with different aryl pendants were evaluated as AChE and BuChE dual inhibitors.

## 2 Methods

### 2.1 General chemistry

**Figure 4:**
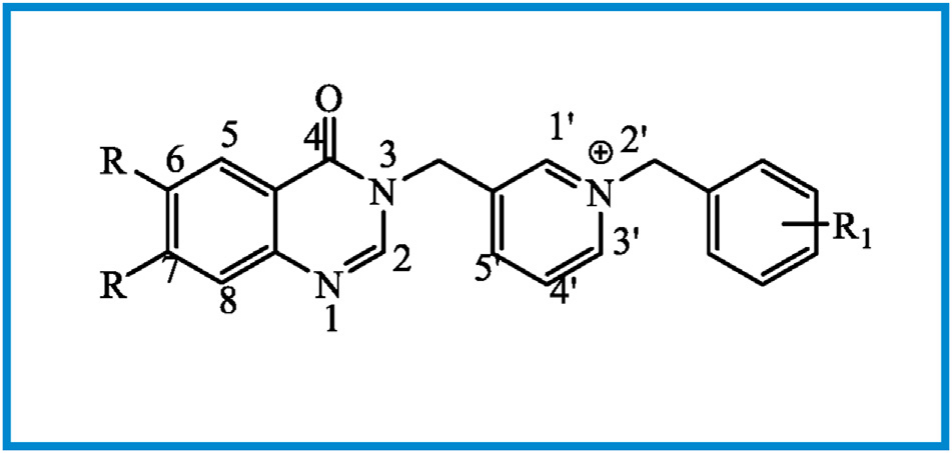
Atom numbering of the BOPs used for ^1^H NMR data.

Sigma Aldrich and Merck provided reagents, and they were utilized as provided without further purification. The ^1^H nuclear magnetic resonance (NMR) spectra for all derivatives were documented by tetramethylsilane (TMS), which is the internal standard on a Bruker FT-500 MHz spectrometer. We presented coupling constants in Hertz (Hz) and expressed chemical shifts as δ (part per million) downfield from TMS as an internal standard (Supplementary). All NMR analyses were carried out at room temperature. Figure 4 indicates the target compounds' atom numbering employed for ^1^H NMR data. Kofler hot stage was used for determining compounds’ melting points. Thin-layer chromatography (TLC) on Merck pre-coated Silica Gel F254 plates was used for routine checking of product mixture and progress of the reaction.

### 2.2 Synthesis

#### 2.2.1 General process of synthesis of (pyridinylmethyl)quinazolin-4(3*H*)-one derivations (9a, b and 12a, b)

We added proper chloromethyl pyridine (1 equiv) to a mixture of quinazolin-4(3*H*)-one derivations (1 equiv) and excess of anhydrous potassium carbonate in 5 ml dry DMF, following by stirring the mix for 4 h under argon at 50 °C. TLC was used for checking the reaction progress. We added 20 ml water and then cooled the mixture, extracting with ethyl acetate (3 × 30 ml). Over Na_2_SO_4_, combined organic extracts were dried out, and the removal of the solvent was performed with reduced pressure. The resulting precipitated solid afforded in a good yield and was used in the next step without further purification.

**3,4-Dimethoxy-3-(pyridine-3-ylmethyl)quinazolin-4(3*H*)-one (9a)**

Starting from 3,4-dimethoxy-quinazolin-4(3*H*)-one (**5**) (1mmol, 0.20gr) and 3-chloromethyl pyridine hydrochloride (1mmol, 0.164gr) compound **9a** was afforded in 71% yield, mp = 142-148 ᵒC, ^1^H NMR (DMSO-*d*_6_, 500 MHz) δ (ppm): 8.65 (s, 1H, H_1⸍_), 8.53 (s, 1H, H_2_) 8.5 (d, *J* = 6.29 Hz, 1H, H_3⸍_), 7.7 (d, *J* = 8.04 Hz, 1H, H_5⸍_), 7.45 (s, 1H, H_5_), 7.37 (t, *J* = 7.15 Hz, 1H, H_4⸍_), 7.15 (s, 1H, H_8_), 5.21 (s, 2H, N-CH_2_-), 3.91 (s, 3H, O-CH_3_), 3.86 (s, 3H, O-CH_3_). ^13^C NMR (DMSO-*d*_6_, 125 MHz) δ (ppm): 160, 152, 148, 147, 146, 144, 140, 135, 132, 125, 115, 107, 106, 57, 57, 46.5.

**3,4-Dimethoxy-3-(pyridine-4-ylmethyl)quinazolin-4(3*H*)-one (9b)**

Starting from 3, 4-dimethoxy-quinazolin-4(3*H*)-one (**5**) (1mmol, 0.20gr) and 4-chloromethyl pyridine hydrochloride (1mmol, 0.164gr) compound **9b** was afforded in 70% yield, mp = 140-145 ᵒC, ^1^H NMR (DMSO-*d*_6_, 500 MHz) δ (ppm): 8.53 (s, 1H, H_2_) 8.4 (d, *J* = 6.1 Hz, 2H, H_2⸍-4⸍_), 7.7 (s, 1H, H_5_), 7.3 (d, *J* = 6.2 Hz, 2H, H_1⸍-5⸍_), 7.15 (s, 1H, H_8_), 5.21 (s, 2H, N-CH_2_-), 3.91 (s, 3H, O-CH_3_), 3.86 (s, 3H, O-CH_3_). ^13^C NMR (DMSO-*d*_6_, 125 MHz) δ (ppm): 161, 153, 149, 147, 145, 144, 140, 122, 114, 108, 106, 56, 56.

**3-(Pyridine-3-ylmethyl)quinazolin-4(3*H*)-one (12a)**

Starting from quinazolin-4(3*H*)-one (**11**) (1mmol, 0.145 gr) and 3-chloromethyl pyridine hydrochloride (1mmol, 0.164gr) compound **12a** was afforded in 65% yield, mp = 130-136 ᵒC,^1^H NMR (DMSO-*d*_6_, 500 MHz) δ (ppm): 8.64 (s, 1H, H_2_), 8.59 (s, 1H, H_1⸍_), 8.37 (d, *J* = 6.2 Hz, 1H, H_3⸍_), 8.16 (d, *J* = 7.4 Hz, 1H, H_5_), 7.86 (d, *J* = 8.04 Hz, 1H, H_5⸍_), 7.7 (m, 2H, H_6,7_), 7.59 (d, *J* = 7.4 Hz, 1H, H_8_), 7.39 (t, *J* = 7.2 Hz, 1H, H_4⸍_), 4.8 (s, 2H, N-CH_2_). ^13^C NMR (DMSO-*d*_6_, 125 MHz) δ (ppm): 162, 150, 149,148.6, 148.2, 137.5, 135, 134, 129, 128, 127.7, 125,121, 47.

**3-(Pyridine-4-ylmethyl)quinazolin-4(3*H*)-one (12b)**

Starting from quinazolin-4(3*H*)-one (**11**) (1mmol, 0.145 gr) and 4-chloromethyl pyridine hydrochloride (1mmol, 0.164gr) compound **12b** was afforded in 65% yield, mp = 128-133 ᵒC, ^1^H NMR (DMSO-*d*_6_, 500 MHz) δ (ppm): 8.64 (s, 1H, H_2_), 8.55 (d, *J* = 6.1 Hz, 2H, H_2⸍-4⸍_), 8.2 (d, *J* = 7.7 Hz, 1H, H_5_), 7.7–7.6 (m, 2H, H_6-7_), 7.5 (d, *J* = 7.7 Hz, 1H, H_8_), 7.3 (d, *J* = 6.2 Hz, 2H, H_1⸍-5⸍_), 5 (s, 2H, N-CH_2_). ^13^C NMR (DMSO-*d*_6_, 125 MHz) δ (ppm): 163, 151, 149, 147, 145, 135, 129, 127, 123, 121, 49.

#### 2.2.2 General procedure for synthesis of 1-(benzyl)-4-((4-oxoquinazolin-3(4*H*)-yl)methyl) pyridin-1-ium chloride and bromide (BOPs)

(Pyridinyl methyl)quinazolin-4(3*H*)-one derivations (1 equiv) was dissolved in dry CH_3_CN (5 ml) under reflux condition. Next, the appropriate benzyl bromides and chlorides derivatives (1.1 equiv) were added dropwise and refluxed for 3-5 h. After completion of the reaction, the solvent was evaporated under reduce pressure. Then, 15 ml n-Hexane was added to the residue and the resulting precipitated solid was collected by filtration, washed with n-hexane and dried. The crystals were further purified if needed by flash chromatography using chloroform–methanol (99:1) as the mobile phase to afford BOP-(1-12).

**1-(3-Bromobenzyl)-3-((4-oxoquinazolin-3(4*H*)-yl) methyl) pyridin-1-ium bromide (BOP-1)**

Yield: 75%; mp: 224–229 ᵒC; ^1^H NMR (DMSO-*d*_6_, 500 MHz) δ (ppm): 9.35 (s, 1H, H_1⸍_), 9.14 (d, *J* = 6.2 Hz, 1H, H_5_), 8.68 (d, *J* = 8.4, 1H, H_3⸍_), 8.63 (s, 1H, H_2_), 8.19–8.13 (m, 2H, H_6-7_), 7.89 (t, *J* = 8.4 Hz, 1H, H_4⸍_), 7.81 (s, 1H, ArH), 7.75 (d, *J* = 8.4 Hz, 1H, H_5⸍_), 7.65 (d, *J* = 7.9 Hz, 1H, ArH), 7.59 (t, *J* = 8 Hz, 1H, ArH), 7.54 (d, *J* = 8 Hz, 1H, ArH), 7.43 (d, *J* = 7.7 1H, H_8_), 5.85 (s, 2H, -N^+^-CH_2_-), 5.41 (s, 2H, -CON-CH_2_-); ^13^C NMR (DMSO-*d*_6_, 125 MHz) δ (ppm): 161.1, 148.4, 146.2, 145, 144.8, 144.6, 144.3, 139, 137, 132.7, 132.12, 131.7, 128.7, 128.4, 122.6, 62, 47.

**1-(4-Chlorobenzyl)-3-((4-oxoquinazolin-3(4*H*)-yl)methyl) pyridin-1-ium chloride (BOP-2)**

Yield: 70%; mp: 221–224 ᵒC; ^1^H NMR (DMSO-*d*_6_, 500 MHz) δ (ppm): 9.45 (s, 1H, H_1⸍_), 9.21 (d, *J* = 7.7 Hz, 1H, H_5_), 8.68 (s, 1H, H_2_), 8.67 (d, *J* = 7.7, 1H, H_3⸍_), 8.17–8.09 (m, 2H, H_6-7_), 7.86 (t, *J* = 7.8 Hz, 1H, H_4⸍_), 7.72 (d, *J* = 8 Hz, 1H, H_8_), 7.61 (d, *J* = 8.27 Hz, 2H, ArH), 7.57 (d, *J* = 7.8 Hz, 1H, H_5⸍_), 7.50 (d, *J* = 8.27 Hz, 2H, ArH), 5.89 (s, 2H, -N + –CH_2_–), 5.42 (s, 2H, –CO–N–CH_2_–); ^13^C NMR (DMSO-*d*_6_, 125 MHz) δ (ppm): 161.1, 148.4, 148.2, 146, 145, 144, 139, 138, 135, 133, 132, 130, 128, 127, 126, 122, 63, 47.

**1-(4-Flourobenzyl)-3-((4-oxoquinazolin-3(4*H*)-yl)methyl) pyridin-1-ium chloride (BOP-3)**

Yield: 76%; mp: 218–223 ᵒC; ^1^H NMR (DMSO-*d*_6_, 500MHz) δ (ppm): 9.45 (s, 1H, H_1⸍_), 9.21 (d, *J* = 7.7 Hz, 1H, H_5_), 8.68 (s, 1H, H_2_), 8.67 (d, *J* = 7.7, 1H, H_3⸍_), 8.17–8.09 (m, 2H, H_6-7_), 7.86 (t, *J* = 7.8 Hz, 1H, H_4⸍_), 7.72 (d, *J* = 8 Hz, 1H, H_8_), 7.61 (m, 2H, ArH), 7.53 (d, *J* = 7.8 Hz, 1H, H_5⸍_), 7.21 (t, *J* = 8.81 Hz, 2H, ArH), 5.89 (s, 2H, -N + –CH_2_–), 5.42 (s, 2H, –CO–N–CH_2_–); ^13^C NMR (DMSO-*d*_6_, 125 MHz) δ (ppm): 161.1,159.9, 148.4, 148.2, 146, 145, 144, 139, 138, 135, 133, 132, 130, 128, 127, 126, 122, 63, 47.

**1-(4-Cyanobenzyl)-3-((4-oxoquinazolin-3(4*H*)-yl)methyl) pyridin-1-ium bromide (BOP-4)**

Yield: 80%; mp: 223–226 ᵒC; ^1^H NMR (DMSO-*d*_6_, 500 MHz) δ (ppm): 9.45 (s, 1H, H_1⸍_), 9.24 (d, *J* = 6.03 Hz, 1H, H_5_), 8.73 (d, *J* = 7.7 Hz, 1H, H_3⸍_), 8.7 (s, 1H, H_2_), 8.2 (t, *J* = 6.2 Hz, 1H, H_6_), 8.13 (d, *J* = 8.06 Hz, 1H, H_5⸍_), 7.94 (d, *J* = 8.14 Hz, 2H, ArH), 7.87 (t, *J* = 8.2 Hz, 1H, H_4⸍_), 7.75 (d, *J* = 8.14 Hz, 2H, ArH), 7.71 (d, *J* = 6.6 Hz, 1H, H_8_), 7.57 (t, *J* = 7.45 Hz, 1H, H_7_), 6 (s, 2H, -N + –CH_2_–), 5.45 (s, 2H, –CO–N–CH_2_–); ^13^C NMR (DMSO-*d*_6_, 125 MHz) δ (ppm): 161.1, 148.4, 148.2, 146, 145, 144, 139, 138, 135, 133, 130, 128, 127, 126, 122, 118, 112, 63, 47.

**1-Benzyl-3-((4-oxoquinazolin-3(4*H*)-yl) methyl) pyridin-1-ium bromide (BOP-5)**

Yield: 78%; mp: 227–231 ᵒC; ^1^H NMR (DMSO-*d*_6_, 500MHz) δ (ppm): 9.49 (s, 1H, H_1⸍_), 9.24 (d, *J* = 6.08 Hz, 1H, H_5_), 8.75 (s, 1H, H_2_), 8.69 (d, *J* = 8 Hz, 1H, H_3⸍_), 8.18 (m, 2H, H_6-7_), 8.12 (d, *J* = 7.8 Hz, 1H, H_5⸍_), 7.87 (t, *J* = 7.8 Hz, 1H, H_4⸍_), 7.73 (d, *J* = 8.2 Hz, 1H, H_8_), 7.57 (d, *J* = 5.35, 2H, ArH), 7.47–7.35 (m, 3H, ArH), 5.92 (s, 2H, -N + –CH_2_–), 5.46 (s, 2H, –CO–N–CH_2_–); ^13^C NMR (DMSO-*d*_6_, 125 MHz) δ (ppm): 161.1, 148.4, 148.2, 146, 145, 144, 139, 138, 135, 133, 130, 128, 127, 126, 125, 122, 63, 47.

**1-(3-Bromobenzyl)-3-((6,7-dimethoxy-4-oxoquinazolin-3(4*H*)-yl)methyl) pyridin-1-ium bromide (BOP-6)**

Yield: 65%; mp: 212–218 ᵒC; ^1^H NMR (DMSO-*d*_6_, 500MHz) δ (ppm): 9.43 (s, 1H, H_1⸍_), 9.21 (d, *J* = 6.1 Hz, 1H, H_3⸍_), 8.64 (d, *J* = 7.8 Hz, 1H, H_5⸍_), 8.58 (s, 1H, H_2_), 8.17 (t, *J* = 6.2 Hz, 1H, H_4⸍_), 7.84 (s, 1H, ArH), 7.62 (d, *J* = 8 Hz, 1H, ArH), 7.58 (d, *J* = 7.87 Hz, 1H, ArH), 7.42 (t, *J* = 7.9 Hz, 1H, ArH), 7.39 (s, 1H, H_5_), 7.15 (s, 1H, H_8_), 5.92 (s, 2H, -N + –CH_2_–), 5.46 (s, 2H, –CO–N–CH_2_–), 3.9 (s, 3H, -O-CH_3_), 3.84 (s, 3H, -O-CH_3_); ^13^C NMR (DMSO-*d*_6_, 125 MHz) δ (ppm): 161.1, 148.4, 146.2, 145, 144.8, 144.6, 144.3, 139, 137, 132.7, 132.12, 131.7, 128.7, 128.4, 122.6, 115, 108, 105, 62, 56.5, 56.2, 47.

**1-(4-Flourobenzyl)-3-((6,7-dimethoxy-4-oxoquinazolin-3(4*H*)-yl)methyl) pyridin-1-ium chloride (BOP-7)**

Yield: 70%; mp: 210–215 ᵒC; ^1^H NMR (DMSO-*d*_6_, 500MHz) δ (ppm): 9.43 (s, 1H, H_1⸍_), 9.20 (d, *J* = 6.1 Hz, 1H, H_3⸍_), 8.61 (d, *J* = 8Hz, 1H, H_5⸍_), 8.54 (s, 1H, H_2_), 8.14 (t, *J* = 6.2 Hz, 1H, H_4⸍_), 7.66 (m, 2H, ArH), 7.39 (s, 1H, H_5_), 7.28 (t, *J* = 8.8 Hz, 2H, ArH), 7.16 (s, 1H, H8), 5.87 (s, 2H, -N + –CH_2_–), 5.3 (s, 2H, –CO–N–CH_2_–), 3.9 (s, 3H, -O-CH_3_), 3.84 (s, 3H, -O-CH_3_); ^13^C NMR (DMSO-*d*_6_, 125 MHz) δ (ppm): 161.1, 159.9, 148.4, 146.2, 145, 144.8, 144.6, 144.3, 139, 137, 130.6, 130, 115.9, 115, 108, 105, 62, 56.5, 56.2, 47.

**1-(4-Chlorobenzyl)-3-((6,7-dimethoxy-4-oxoquinazolin-3(4*H*)-yl)methyl) pyridin-1-ium chloride (BOP-8)**

Yield: 65%; mp: 210–215 ᵒC; ^1^H NMR (DMSO-*d*_6_, 500MHz) δ (ppm): 9.43 (s, 1H, H_1⸍_), 9.20 (d, *J* = 6.1 Hz, 1H, H_3⸍_), 8.61 (d, *J* = 8 Hz, 1H, H_5⸍_), 8.54 (s, 1H, H_2_), 8.14 (t, *J* = 6.2 Hz, 1H, H_4⸍_), 7.6 (d, *J* = 8.3 Hz, 2H, ArH), 7.51 (d, *J* = 8.3 Hz, 2H, ArH), 7.4 (s, 1H, H_5_), 7.1 (s, 1H, H_8_), 5.87 (s, 2H, -N + –CH_2_–), 5.3 (s, 2H, –CO–N–CH_2_–), 3.9 (s, 3H, -O-CH_3_), 3.84 (s, 3H, -O-CH_3_); ^13^C NMR (DMSO-*d*_6_, 125 MHz) δ (ppm): 161.1, 148.4, 146.2, 145, 144.8, 144.6, 144.3, 139, 137, 132, 131.3, 130.6, 130, 128, 115, 108, 105, 62, 56.5, 56.2, 47.

**1-(4-Cyanobenzyl)-3-((6,7-dimethoxy-4-oxoquinazolin-3(4*H*)-yl)methyl) pyridin-1-ium bromide (BOP-9)**

Yield: 75%; mp: 216–219 ᵒC; ^1^H NMR (DMSO-*d*_6_, 500MHz) δ (ppm): 9.43 (s, 1H, H_1⸍_), 9.20 (d, *J* = 6.1 Hz, 1H, H_3⸍_), 8.61 (d, *J* = 8 Hz, 1H, H_5⸍_), 8.54 (s, 1H, H_2_), 8.14 (t, *J* = 6.2 Hz, 1H, H_4⸍_), 7.6 (d, *J* = 8.3 Hz, 2H, ArH), 7.51 (d, *J* = 8.3 Hz, 2H, ArH), 7.4 (s, 1H, H_5_), 7.1 (s, 1H, H_8_), 5.87 (s, 2H, -N + –CH_2_–), 5.3 (s, 2H, –CO–N–CH_2_–), 3.9 (s, 3H, -O-CH_3_), 3.84 (s, 3H, -O-CH_3_); ^13^C NMR (DMSO-*d*_6_, 125 MHz) δ (ppm): 161.1, 148.4, 146.2, 145, 144.8, 144.6, 144.3, 139, 137, 133, 130, 128, 118, 112, 108, 105, 62, 56.5, 56.2, 47.

**1-Benzyl-3-((6,7-dimethoxy-4-oxoquinazolin-3(4*H*)-yl)methyl) pyridin-1-ium bromide (BOP-10)**

Yield: 65%; mp: 210–215 ᵒC; ^1^H NMR (DMSO-*d*_6_, 500MHz) δ (ppm): 9.43 (s, 1H, H_1⸍_), 9.20 (d, *J* = 6.1 Hz, 1H, H_3⸍_), 8.61 (d, *J* = 8 Hz, 1H, H_5⸍_), 8.54 (s, 1H, H_2_), 8.14 (t, *J* = 6.2 Hz, 1H, H_4⸍_), 7.66 (m, 2H, ArH), 7.39 (s, 1H, H_5_), 7.28 (t, *J* = 8.8 Hz, 2H, ArH), 7.16 (s, 1H, H_8_), 5.87 (s, 2H, -N + –CH_2_–), 5.3 (s, 2H, –CO–N–CH_2_–), 3.9 (s, 3H, -O-CH_3_), 3.84 (s, 3H, -O-CH_3_); ^13^C NMR (DMSO-*d*_6_, 125 MHz) δ (ppm): 161.1, 159.9, 148.4, 146.2, 145, 144.8, 144.6, 144.3, 138, 134, 129.8, 129.5, 129.2, 128.6, 115.1, 108, 105, 62, 56.5, 56.2, 47.

### 2.3 Cholinesterase inhibitory assay

The Ellman's approach was used for evaluating BuChE and AChE inhibitory functions of derivatives [22]. Fluka provided potassium hydroxide, sodium hydrogen carbonate, potassium dihydrogen phosphate, and dipotassium hydrogen phosphate. Sigma-Aldrich (Steinheim, Germany) provided BuChE (E.C.3.1.1.8, from equine serum), Electrical (Torpedo californica) AChE (type VI-S), acetylthiocholine iodide, 5,5⸍-dithiobis [2-nitrobenzoic acid] (DTNB), Donepezil hydrochloride as the reference drug, and butyrylthiocholine iodide.

In order to set the derivatives’ stock solutions, they were dissolved in the dimethyl sulfoxide (DMSO). Then, we diluted them in the absolute ethanol for obtaining 3 varying concentrations for assay. The experiments were done in triplicate for each concentration. The assay solution included 60 mL DTNB, 2mL of phosphate buffer (0.1M, pH = 8), 20 mL of 5 IU/mL butryl cholinesterase solution, and 30 mL of inhibitor. Afterwards, the product was pre-incubated at 25 ᵒC for 10 min. Then, 20 mL of butyrylthiocholine iodide was added as the substrate to the 24 wells for starting the reaction. A Synergy HTX multimode plate reader was employed for recording the changes in the absorbance for 5 min at 412 nm for 5 min. For justifying non-enzymatic reaction, we conducted assays with a blank having all components except ButChE.

Calculation of the IC_50_ values was done graphically using Microsoft Excel 2019 from a concentration inhibition curve for the derivatives. Moreover, AChE took the same assay for obtaining the derivatives’ anti-AChE activity.

### 2.4 Docking studies

Using www.rcsb.org, the receptor pdb text file AChE with donepezil bearing PDB code (1EVE) and BuChE (6QAA) were taken. Then, PMV 1.5.6 was used for removing water and the complex ligand. The ligand's atomic co-ordinates (BOP-1 and BOP-8) were drawn by Hyperchem 8.1.10. The addition of polar hydrogen and electric charges were performed in the AutoDock program. The AutoDock parameters for AChE docking were established as following: Grid Box size 60 × 60 × 60, with the center of 5.077–65.107–55.746 as x, y, and z, respectively, and spacing of 0.375 Å. For BuChE docking process a Grid Box size 30 × 30 × 30, with the center of 17.0–41.10–39.0 as x, y, and z, respectively, and spacing of 0.375 Å were used. We set other parameters as default. The ranking of the measured geometries was based on binding free energies, and for further analysis, the best positions were selected. The Discovery Studio software was used for conducting molecular visualization.

## 3 Results and discussion

### 3.1 Chemistry

**Figure 5:**
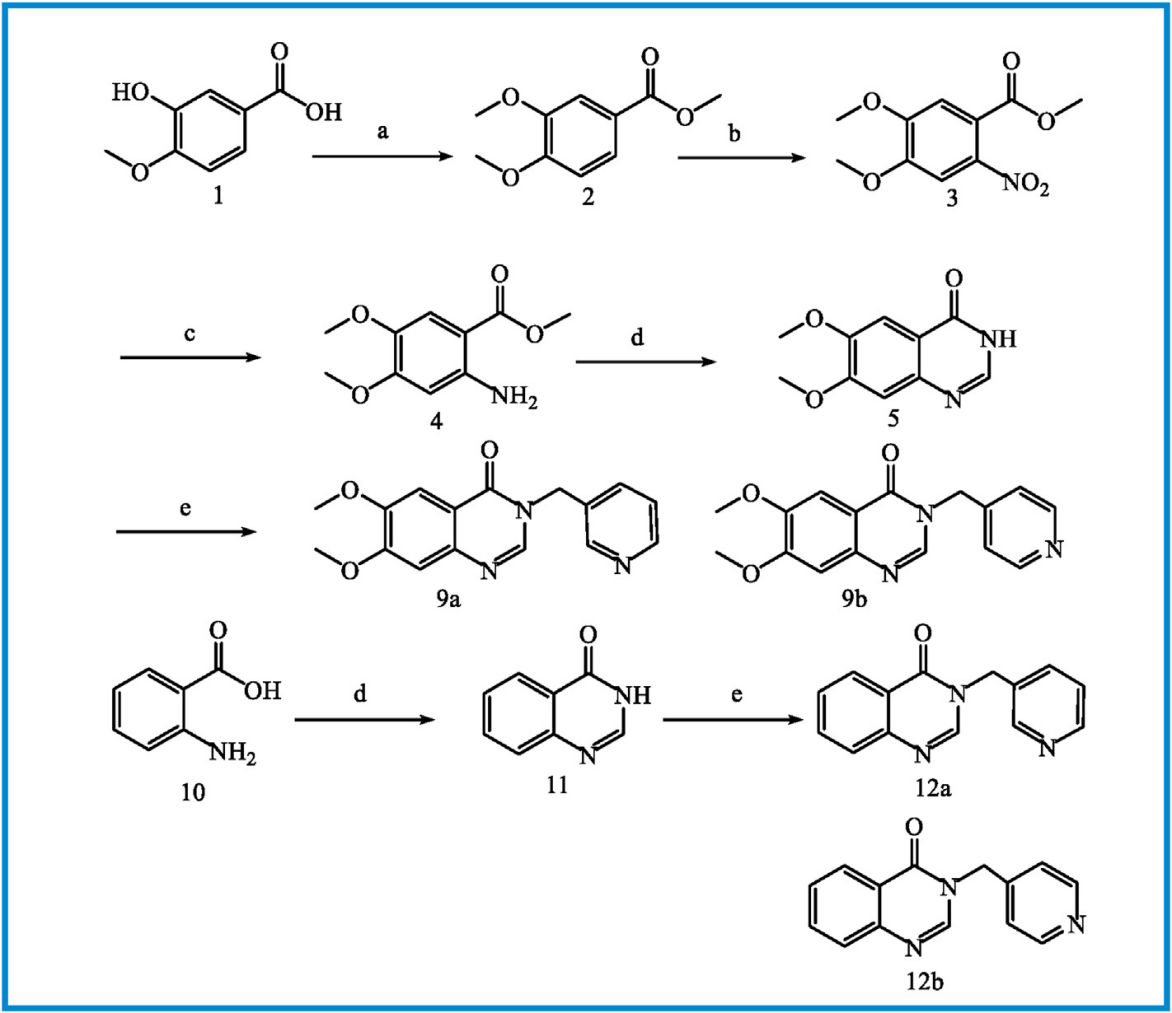
Reagents and conditions: (a) MeI, DMF, rt, overnight; (b) HNO_3_; (c) SnCl_2_, HCl, 0 °C to rt, 3h; (d) formamidine acetate, DMF, 100 °C, 16h; (e) 3&4-chloromethyl pyridine, K_2_CO_3_, DMF, 50 °C, 4 h.

**Figure 6:**
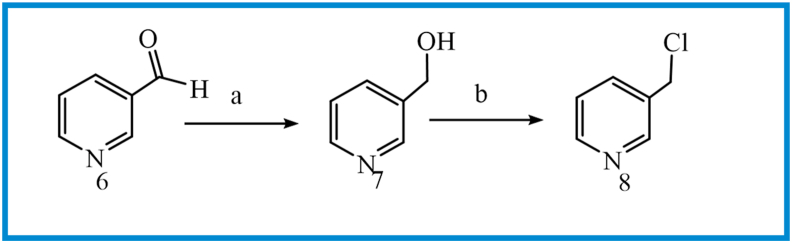
Reagents and conditions: (a) NaBH_4_, MeOH, 0 °C to rt, 3h; (b) SOCl_2_, CH_2_Cl_2_, 0 °C to reflux, 3h.

**Figure 7:**
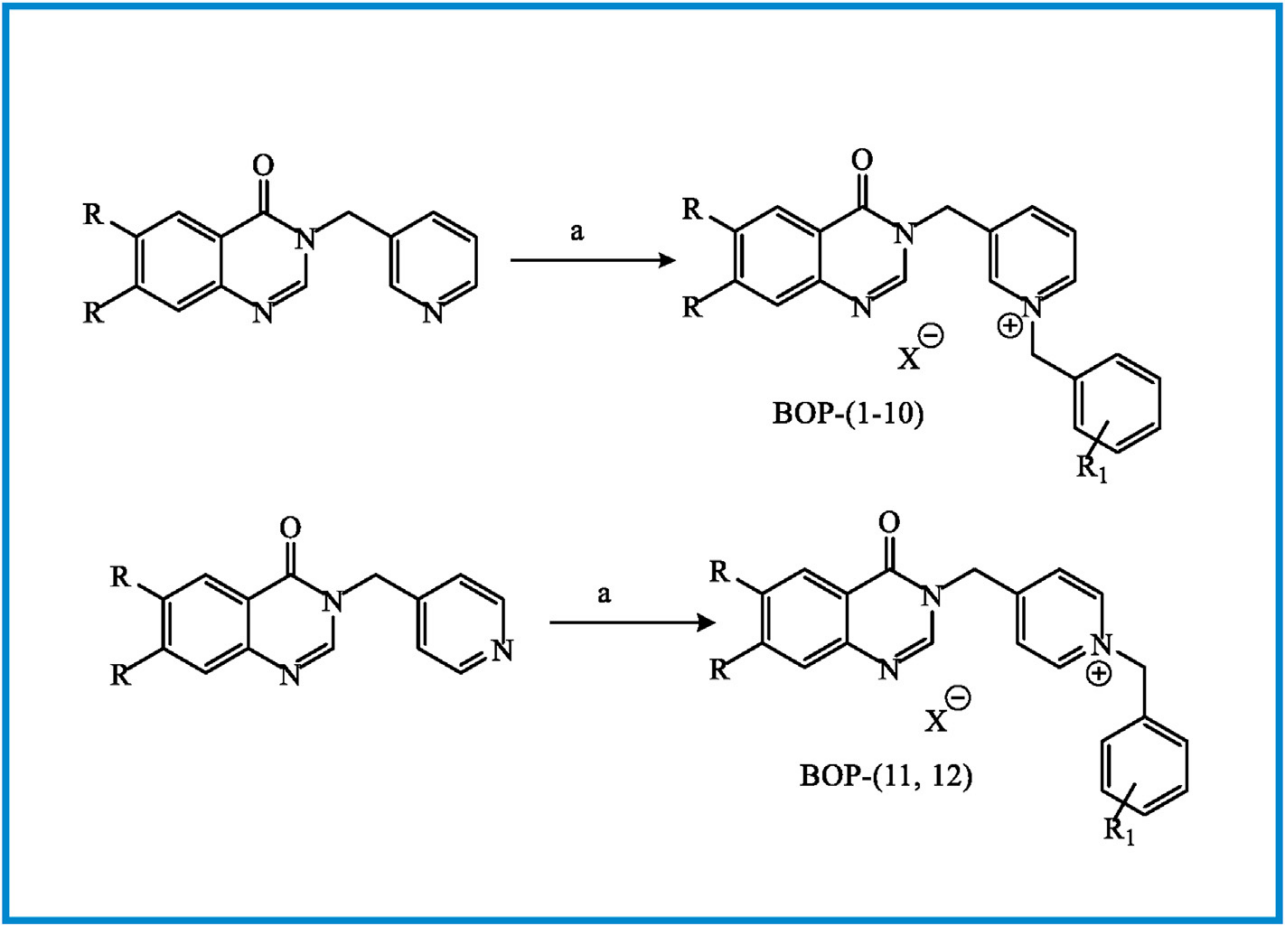
Reagents and conditions: (a) benzyl halide derivatives, CH_3_CN, reflux, 3–5 h.

The synthetic path for preparing BOPs is summarized in Figures 5, 6, 7. Briefly, the reaction of 3-hydroxy-4-methoxy benzoic acid (**1**) with iodomethane in DMF furnished methyl 3,4-dimethoxy benzoat (**2**). Nitration and subsequent reduction of compound **2** afforded compound **4**. The 6,7-dimethoxyquinazolin-4(3*H*)-one (**5**) was prepared by ring closure of **4** in the presence of formamidine acetate. The intermediates **5** or **11** reacted with 3 or 4-(chloromethyl)pyridine (Figure 6) in DMF in the presence of K_2_CO_3_ to obtain compounds **9a, b** and **12a, b,** respectively. Final compounds (BOPs) were gained by adding appropriate benzyl halide derivatives to complexes **9a, b** and **12a, b** in refluxing dry acetonitrile.

### 3.2 Cholinesterase inhibition assay

With modified Ellman's method, anticholinesterase activities of BOPs and donepezil hydrochloride as the reference compound were determined. As observed in Table 1, anticholinesterase activities of synthesized complexes given by IC_50_ values are presented. According to Table 1, BOPs could be categorized into two group: 1) having methoxy group substituted on oxoquinazoline ring 2) without methoxy group on oxoquinazoline ring. For both groups, anti-BuChE and anti-AChE effects could be altered by the replacement of different benzyl halides linked to 3 or 4-(chloromethyl)pyridine moieties. In group-1 of tested compounds (BOP-1-5), BOP-1 having bromine atom on C-3 site of benzyl group showed the strongest AChE inhibitory effect (IC_50_ = 5.90 ± 0.07μM). Altering the site and substitution of chlorine atom on C-4 position led to the compound BOP-2 with lower AChE inhibitory activity (IC_50_ = 47.14 ± 0.48μM). Also, supplanting the chlorine atom by the fluorine on BOP-3 or CN on BOP-4 resulted in derivatives with very low activities. Although with the removal of this group on benzyl ring the AChE inhibitory raised for compound BOP-5 (IC_50_ = 41.21 ± 0.62μM), compound BOP-1 remained the best AChE inhibitor in this group of compounds. For BuChE inhibitory activities, compounds in group-1 revealed similar manner to the AChE inhibitory activity and compound BOP-3 with fluorine atom on C-4 showed better result in comparison to its AChE inhibition activity (AChE IC_50_ > 100 μM, BuChE IC_50_ = 70.43 ± 0.41μM). For BOP-5 with no substitution on benzyl ring, BuChE inhibition reached the IC_50_ of 12.63 ± 0.11μM, lower compared with AChE which enjoys an IC_50_ of 41.21 ± 0.62μM. In group-1, the BuChE inhibitory activities were more than AChE inhibition except for BOP-1 with slightly better AChE activity (AChE IC_50_ = 5.90 ± 0.07μM, BuChE IC_50_ = 6.76 ± 0.04μM) that was the best compound in this group.

**Table 1:** Cholinesterase inhibition IC_50_ average ±SD of BOPs and donepezil hydrochloride.


Compound	R	R_1_	X	AChE Inhibition [IC_50_ (μM)]	BuChE Inhibition [IC_50_ (μM)]
BOP-1	H	3-Br	Br	**5.90 ± 0.07**	**6.76 ± 0.04**
BOP-2	H	4-Cl	Cl	47.14 ± 0.48	29.04 ± 0.43
BOP-3	H	4-F	Cl	>100	70.43 ± 0.41
BOP-4	H	4-CN	Br	>100	>100
BOP-5	H	H	Br	41.21 ± 0.62	12.63 ± 0.11
BOP-6	OMe	3-Br	Br	6.77 ± 0.24	8.77 ± 0.11
BOP-7	OMe	4-F	Cl	21.92 ± 0.2	>100
BOP-8	OMe	4-Cl	Cl	**1.11 ± 0.09**	>100
BOP-9	OMe	4-CN	Br	>100	>100
BOP-10	OMe	H	Br	10.08 ± 0.28	18.84 ± 0.34
BOP-11	OMe	4-CN	Br	>100	>100
BOP-12	OMe	4-Cl	Cl	>100	>100
Donepezil	-	-	-	0.079 ± 0.002	5.19 ± 0.38

Bold value signifies the most active compounds related IC_50_ values.

In a second group containing methoxy substitution on oxoquinazolin ring (BOP-6-12), the best compound was BOP-8 with a chlorine atom on C-4 position which showed promising potency as AChE inhibitor (IC_50_ = 1.11 ± 0.09μM). However, BOP-8 had no inhibitory activity against BuChE, which indicate that this compound is a good selective AChE inhibitor. The replacement of chlorine with hydrogen, fluorine and CN in position C-4 caused depletion of inhibitory activity for AChE in compounds BOP-10, BOP-7 and BOP-9 with IC_50_ = 10.08 ± 0.28μM, IC_50_ = 21.92 ± 0.2μM and IC_50_ > 100 μM, respectively. The BOP-6 with 3-Bromo benzyl ring revealed an intermediate potency among other compounds in this group (IC_50_ = 6.77 ± 0.24μM). For compounds BOP-6 to BOP-12 lower inhibitory effect on BuChE was observed (except BOP-6 and BOP-10 with intermediate IC_50_ values of 8.77 ± 0.1μM, 18.84 ± 0.34μM respectively). Two of our compounds with 4-(methyl)pyridine moiety (BOP-11 and BOP-12) showed lower inhibitory activities both for AChE and BuChE in comparison with other compounds containing 3-(methyl)pyridine group. According to the observed results, group-1 had a higher activity for BuChE inhibition. On the other hand, group 2 with the substituted methoxy groups had better results for AChE inhibition. It is worthwhile to note that, BOPs with 3-methylpyridine moieties showed superior activities than 4-methylpyridine derivatives and the electron-withdrawing CN group decreased the inhibitory activity. Finally, compound BOP-1 with AChE IC_50_ = 5.90 ± 0.07μM and BuChE IC_50_ = 6.76 ± 0.04μM had the most dual inhibitory activity and compound BOP-8 with AChE IC_50_ = 1.11 ± 0.09 μM was the strongest complex among tested compounds against AChE.

### 3.3 Screening of physicochemical characteristics

The Lipinski's rule of five is usually used to estimate drug-likeness or decide if a compound with a certain biological activity has properties that would make it a potential active drug in humans. We calculated the pharmacokinetic profile and possible violations of the rule of five of the most active compounds BOP-1 and BOP-8 with Swiss ADME web-based tool [23] and presented in Table 2. As shown by the data calculated, both of compounds have suitable properties without violating the Lipinski's rule of five. Considering the fact that the majority of the biologically active complexes known as drug candidates have not more than one violation of the Lipinski's criteria. Both BOP-1 and BOP-8 pursued the criteria and, hence, they can be regarded as drug candidates.

**Table 2:** Swiss ADME pharmacokinetics prediction for the compounds BOP-1 and BOP-8.

Compound	MW (g/mol)	Bioavailability Score^a^	Solubility^b^	Log P^c^	Drug likeness
BOP-1	487.19	0.55	Moderately soluble	4.7	Yes
BOP-8	458.34	0.55	Moderately soluble	4.4	Yes

a Abbott bioavailability score: probability of F>10% in rat.

b Solubility class: log S scale.

c Calculated by XLOGP program version 3.2.2.

### 3.4 Docking studies

Docking study was accompanied for investigating the binding state of the most active complexes BOP-8 and BOP-1 in the active position of BuChE (PDB:6QAA) and AChE (PDB: 1EVE), which was performed by using Autodock 4.

**Figure 8:**
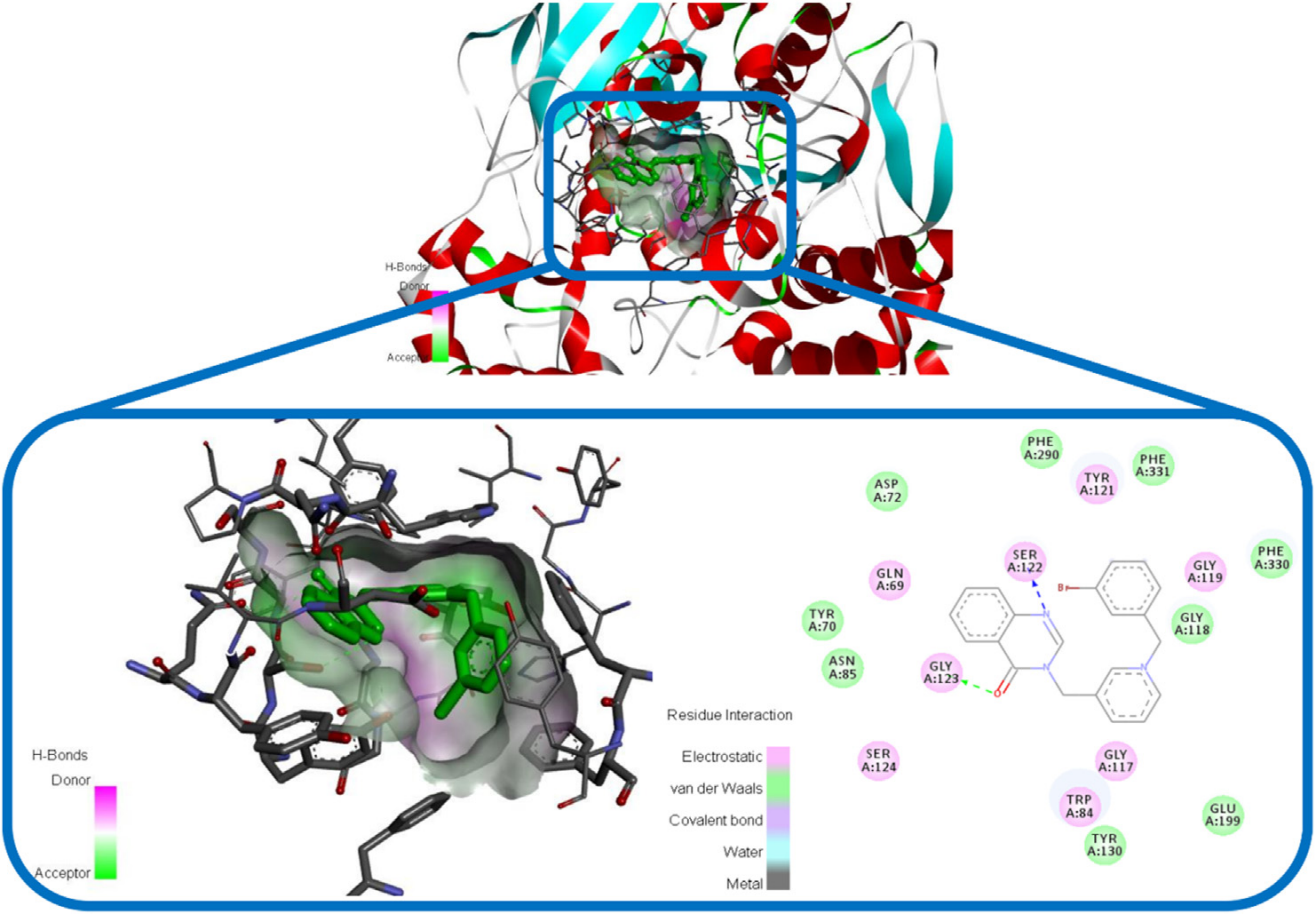
The binding mode of compound BOP-1 in the active site of AChE.

**Figure 9:**
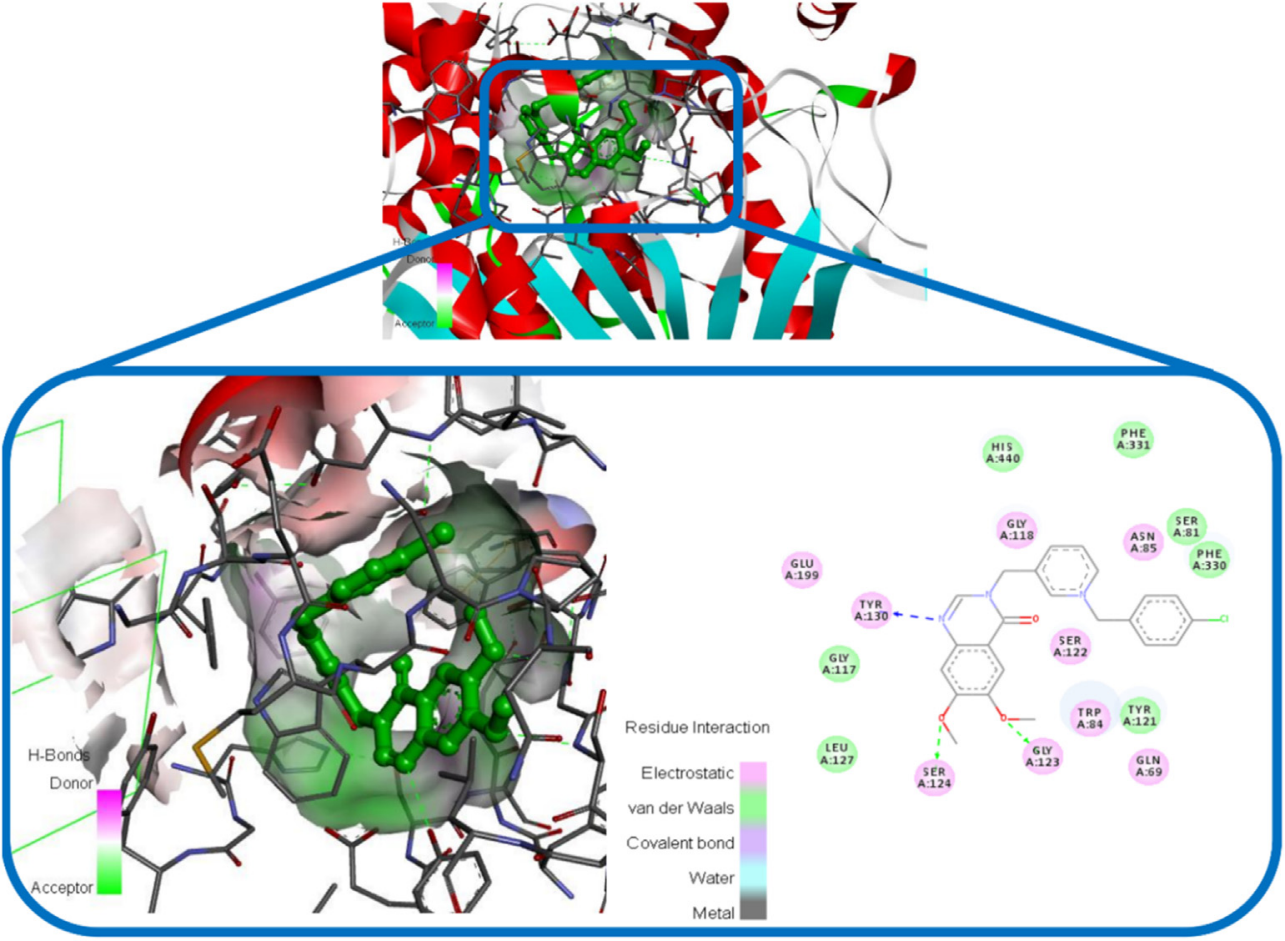
The binding mode of compound BOP-8 in the active site of AChE.

As can be seen in Figure 8 and Figure 9, the orientation of compound BOP-1 and BOP-8 were studied in the active position of AChE. The results showed that BOP-1 and BOP-8 were strongly bound with the optimal conformation of AChE, and their binding energies reached -10.75 kcal∗mol-1 and -10.26 kcal∗mol-1. According to the interaction mode of BOP-1 (Figure 8), the nitrogen atom of quinazolinon aligned toward SER 122 residues via an H-bond interaction and Oxygen atom of carbonyl group interacted with Gly123 through another H-bond interaction.

In case of compound BOP-8 as shown in Figure 9, nitrogen atom of quinazolinon aligned toward Tyr130 residues via an H-bond interaction and methoxy groups interacted with Gly123 and SER 124 through H-bond interactions. These tree key H-bond interactions demonstrated the high inhibitory potency of compound BOP-8 for AChE.

**Figure 10:**
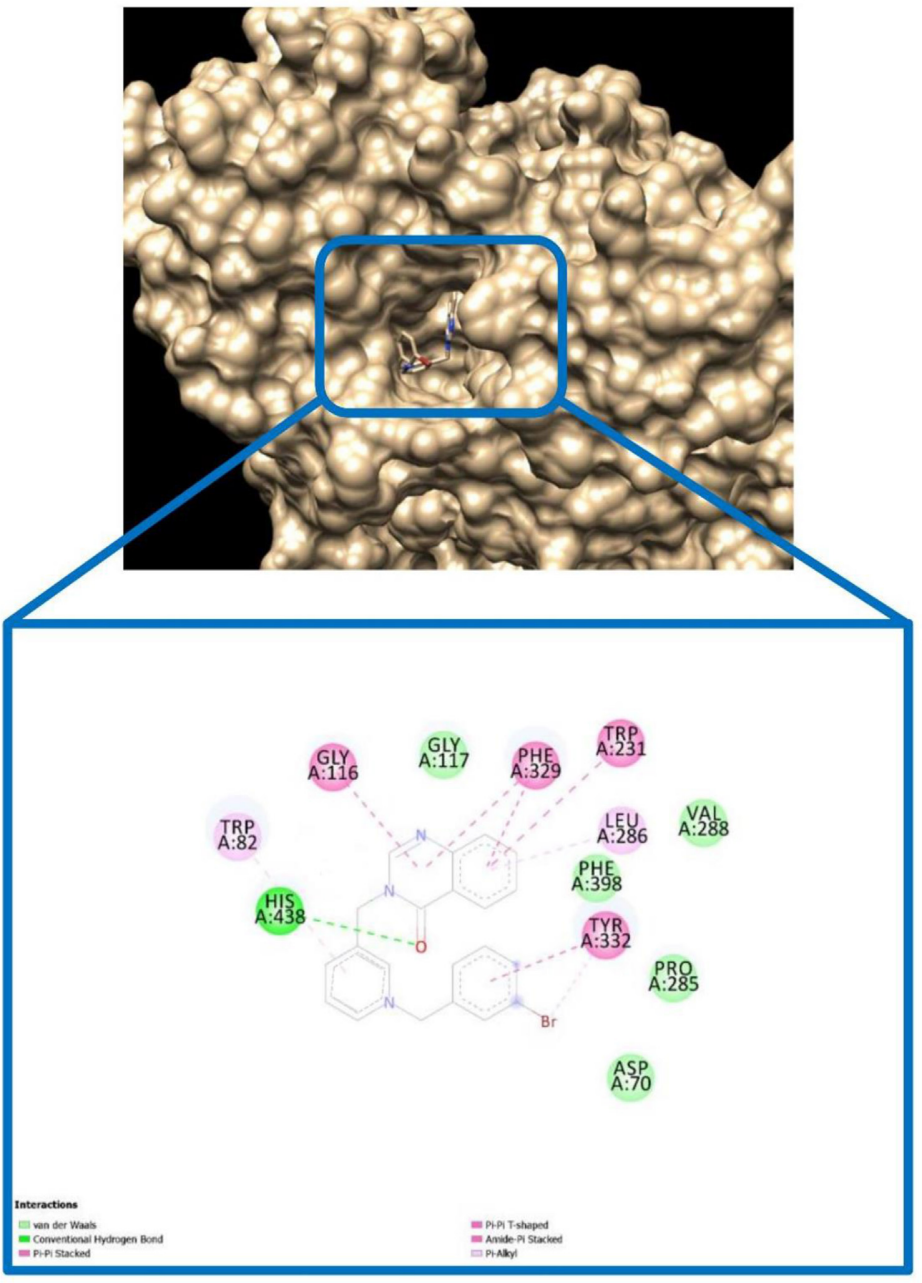
The binding mode of compound BOP-1 in the active site of BuChE.

For compound BOP-1 in complex with BuChE (Figure 10), a conventional H-bond of carbonyl oxygen with HIS-438 and also pi-pi stacking interactions for phenyl ring of quinazolinon and benzyl ring with several active site residues was observed.

**Figure 11:**
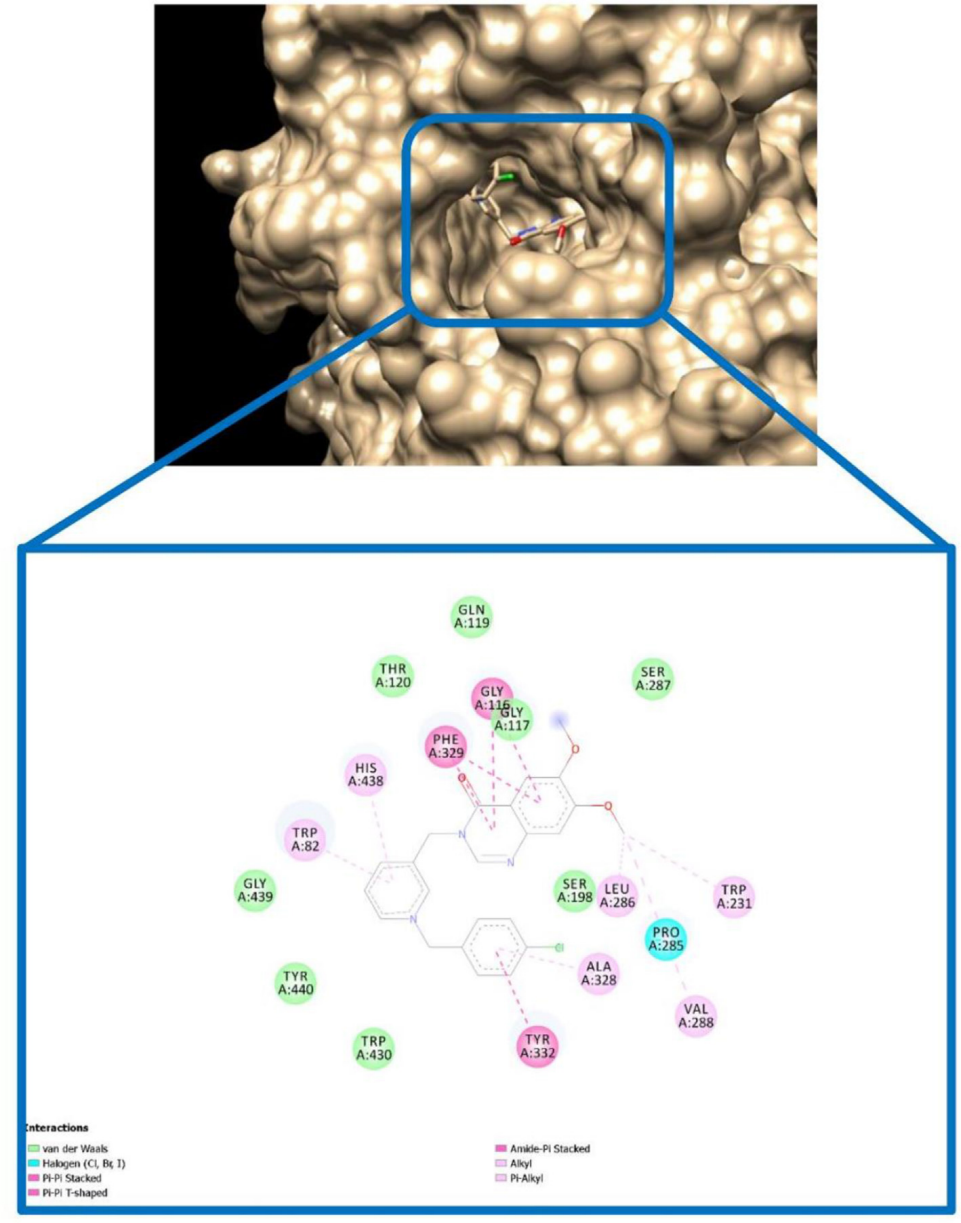
The binding mode of compound BOP-8 in the active site of BuChE.

We also conducted docking to study interactions of compound BOP-8 as a selective inhibitor of AChE in the active site of BuChE. As shown in Figure 11, there were no major interactions with active site residues in comparison to AChE binding pattern especially lack of H-bonds and this is in line with in vitro assay for compound BOP-8 as a weak BuChE inhibitor.

## 4 Conclusion

We synthesized and assessed novel oxoquinazolin-benzyl pyridinuim hybrids as strong inhibitors of cholinesterase. According to research findings, it was proved that moderate to good AChEI and BuChEI activity of synthesized BOPs, among them compound BOP-1 showed the best anti-AChE and anti-BuChE dual effect with IC_50_ values of 5.90 ± 0.07 and 6.76 ± 0.04μM, respectively. Also, compound BOP-8 possesses selective and potent AChEI activity with IC_50_ values of 1.11 ± 0.09 μM and no inhibition on BuChE, which confirmed with docking studies. Furthermore, the pharmacokinetic properties of BOP-1 and BOP-8 were supported by the Lipinski rules of 5. In general, the present research showed a novel strong dual inhibitor of AChE and BuChE (BOP-1) and a new selective potent anti-AChE agent (BOP-8) with a potential therapeutic advantage and further research value for the treatment of AD.

## Declarations

### Author contribution statement

Samaneh Zarei, Maryam Firouzi: Performed the experiments.

Mohammad Shafiei: Performed the experiments; Wrote the paper.

Loghman Firoozpour, Tahmineh Akbarzadeh: Contributed reagents, materials, analysis tools or data.

Kouros Divsalar, Ali Asadipour: Analyzed and interpreted the data.

Alireza Foroumadi: Conceived and designed the experiments.

### Funding statement

This work was supported by Neuroscience Research Center, Institute of Neuropharmacology, 10.13039/501100004621Kerman University of Medical Sciences (99-33).

### Data availability statement

Data included in article/supplementary material/referenced in article.

### Declaration of interests statement

The authors declare no conflict of interest.

### Additional information

No additional information is available for this paper.
