# Supplementary material for: Design, synthesis and biological assessment of new 1-benzyl-4-((4-oxoquinazolin-3(4H)-yl)methyl) pyridin-1-ium derivatives (BOPs) as potential dual inhibitors of acetylcholinesterase and butyrylcholinesterase
Source: Heliyon. 2021 Apr 8;7(4):e06683. doi: 10.1016/j.heliyon.2021.e06683 (PMC8045006; doi:10.1016/j.heliyon.2021.e06683)

**Supplementary**

**Design, synthesis and biological assessment of new 1-benzyl-4-((4-oxoquinazolin-3(4H)-yl)methyl) pyridin-1-ium derivatives (BOPs) as potential dual inhibitors of acetylcholinesterase and butyrylcholinesterase**

Samaneh Zareia, Mohammad Shafieib, Maryam Firouzia, Loghman Firoozpoura, Kouros Divsalarc, Ali Asadipourd, Tahmineh Akbarzadeha, Alireza Foroumadia, c *

^a^ Department of Medicinal Chemistry, Faculty of Pharmacy and Drug Design & Development Research Center, The Institute of Pharmaceutical Sciences (TIPS), Tehran University of Medical Sciences, Tehran, Iran

^b^ Department of Medicinal Chemistry, Faculty of Pharmacy, Birjand University of Medical Sciences, Birjand, Iran

^c^ Neuroscience Research Center, Institute of Neuropharmacology, Kerman University of Medical Sciences, Kerman, Iran

^d^ Pharmaceutical Sciences and Cosmetic Products Research Center, Kerman University of Medical Sciences, Kerman, Iran

* Corresponding author, E mail: aforoumadi@yahoo.com, <Tel:0098-21-66954708-Fax:0098-21-66461178>

**^1^H-NMR and ^13^C-NMR Spectral of synthetic compounds (BOPs) ……….………... 2-11**

^1^H-NMR and ^13^C-NMR Spectral of synthetic compounds:

BOP-1


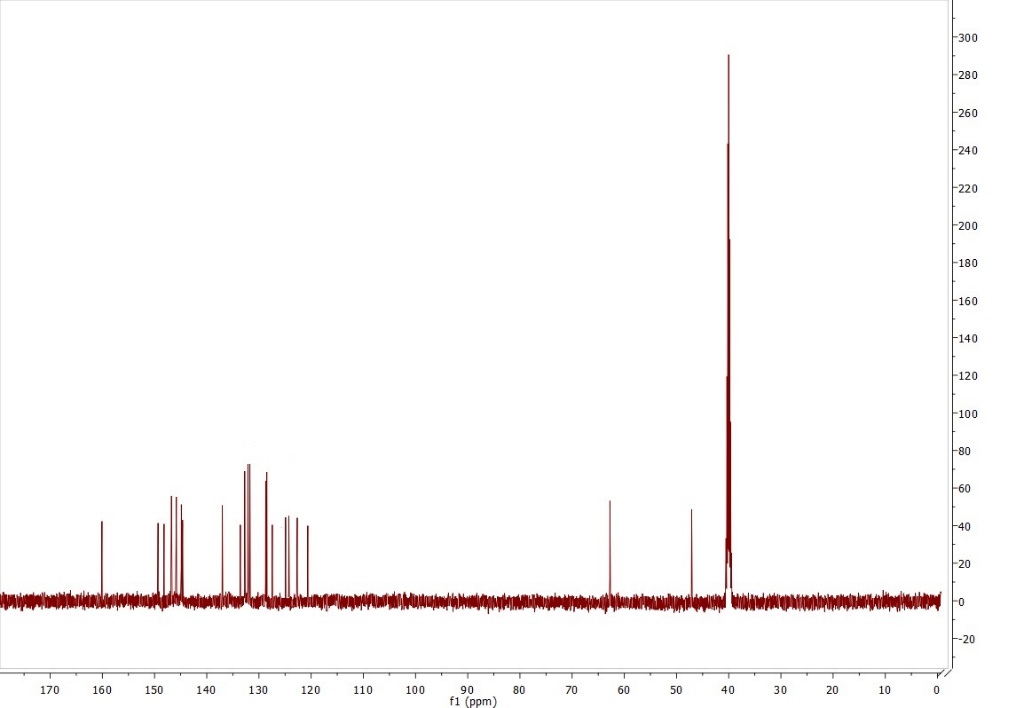

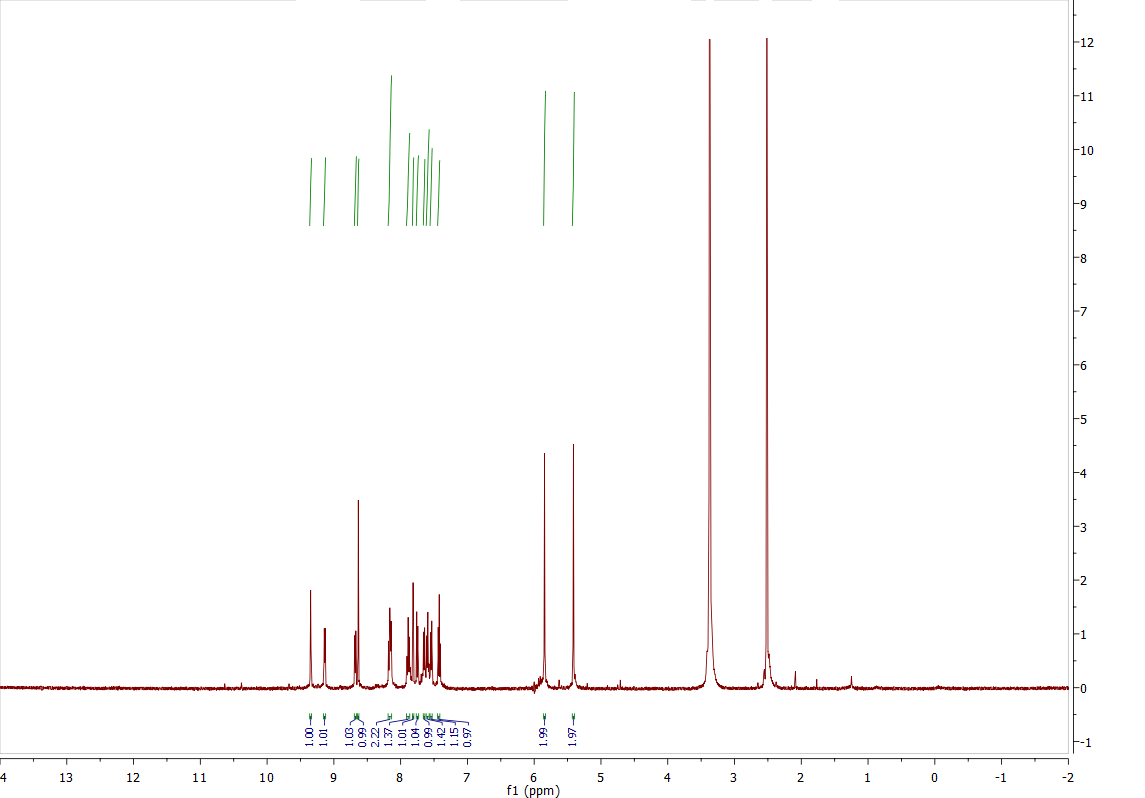


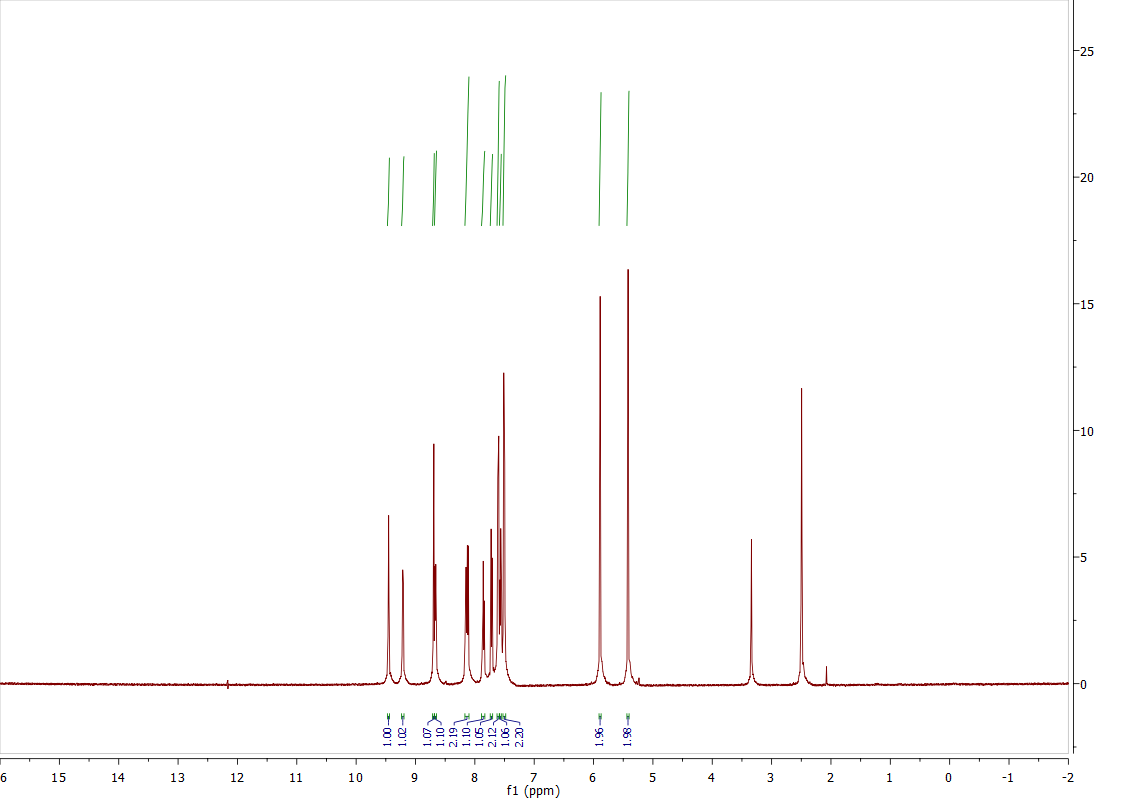
BOP-2


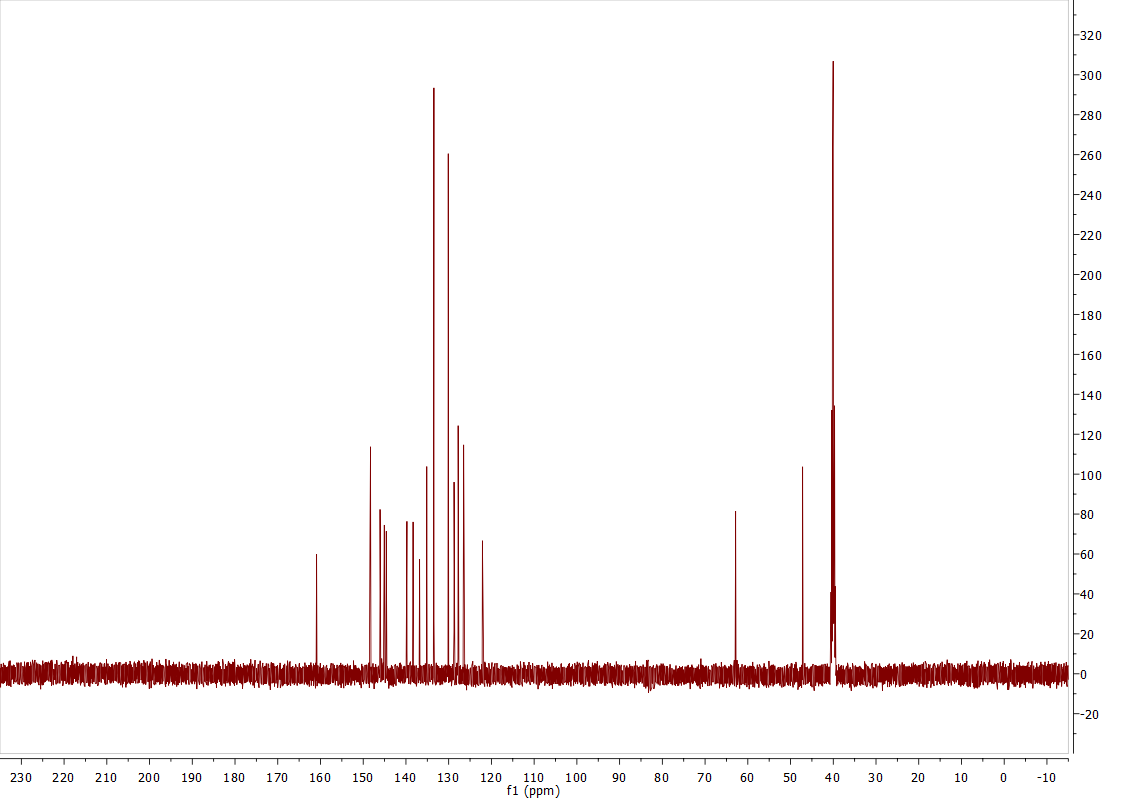


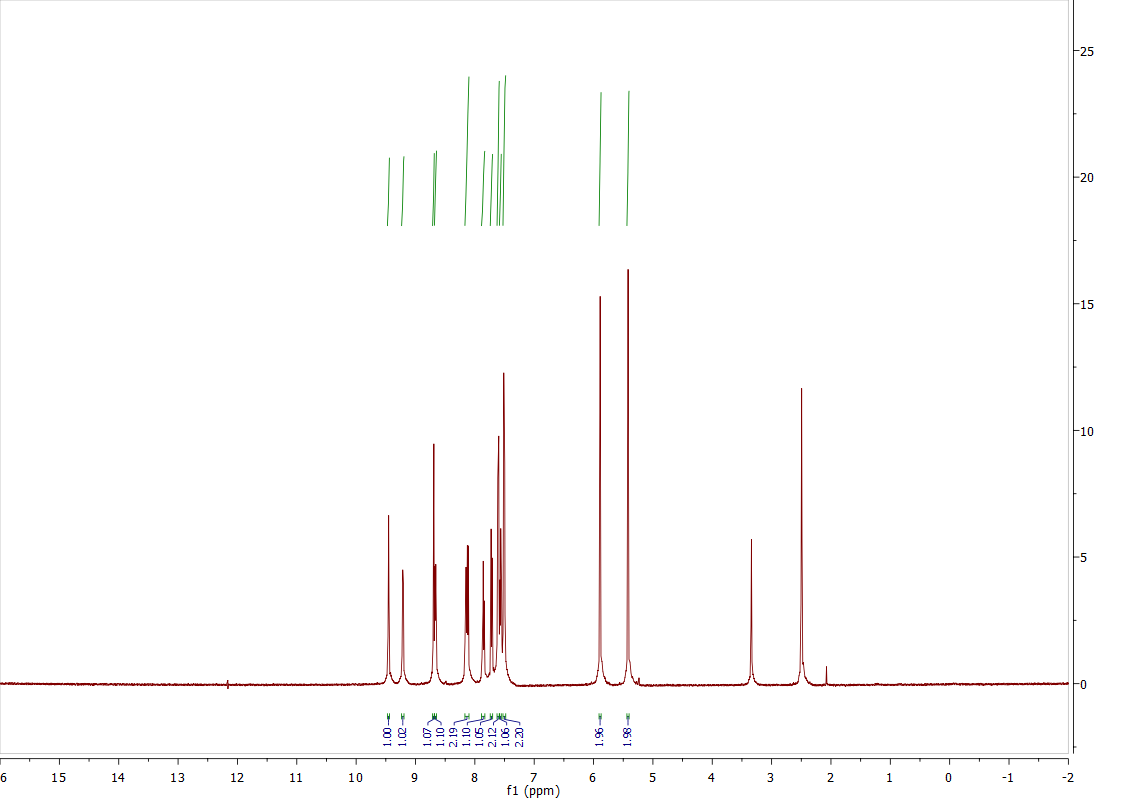
BOP-3


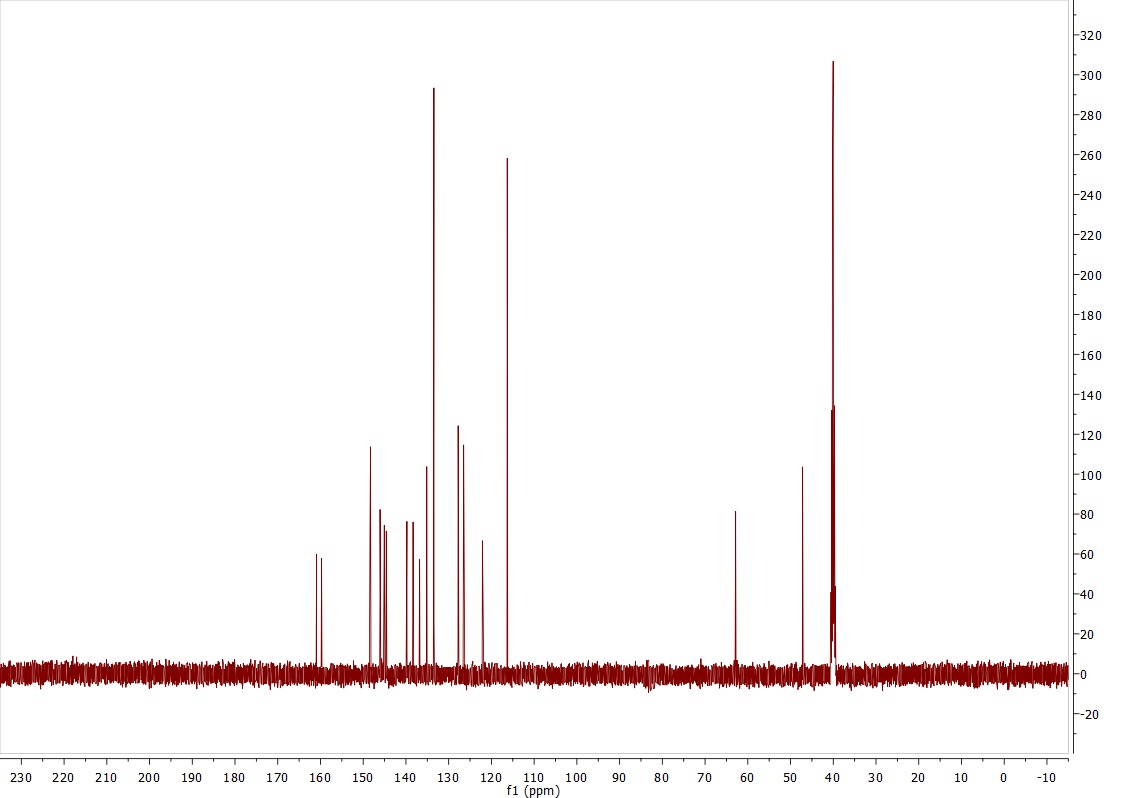


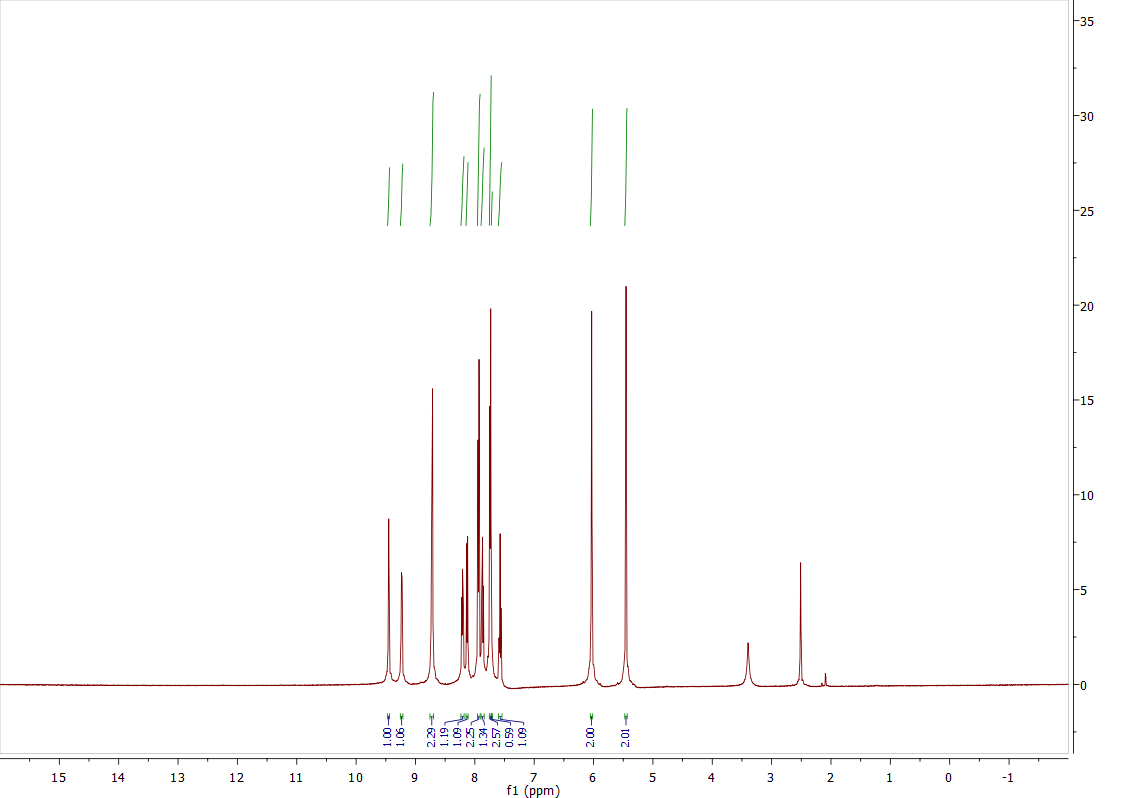
BOP_4


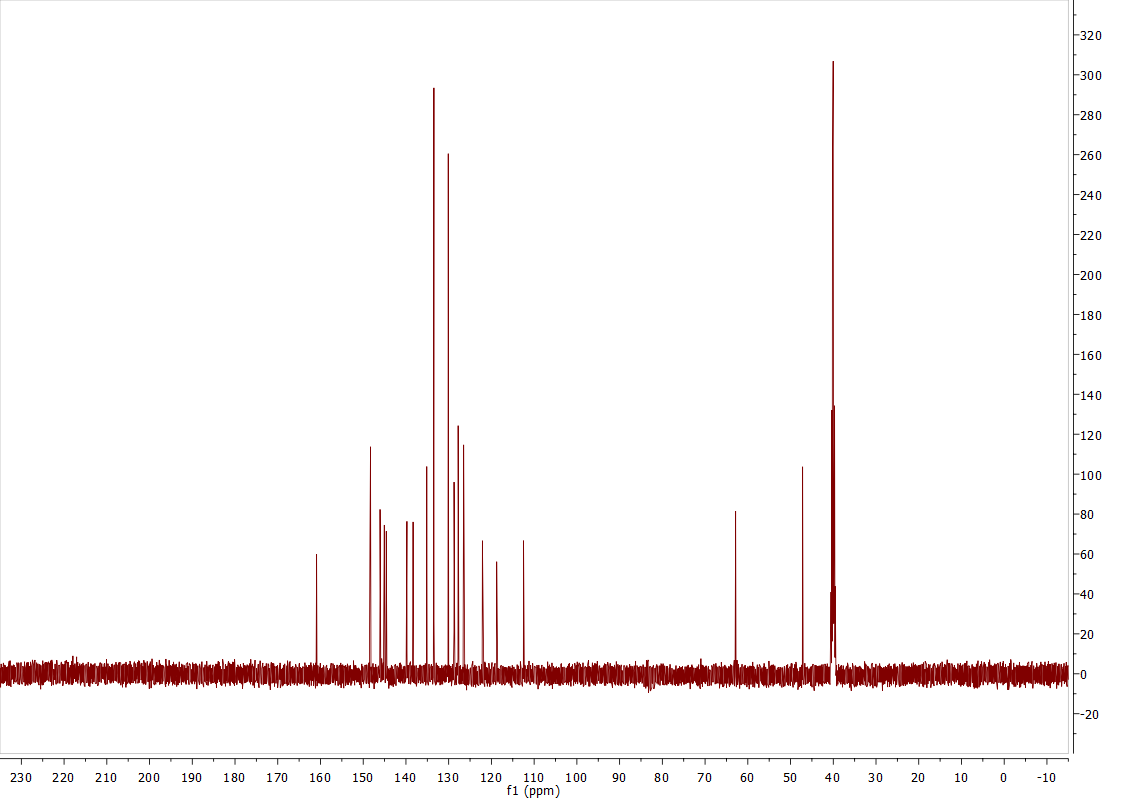


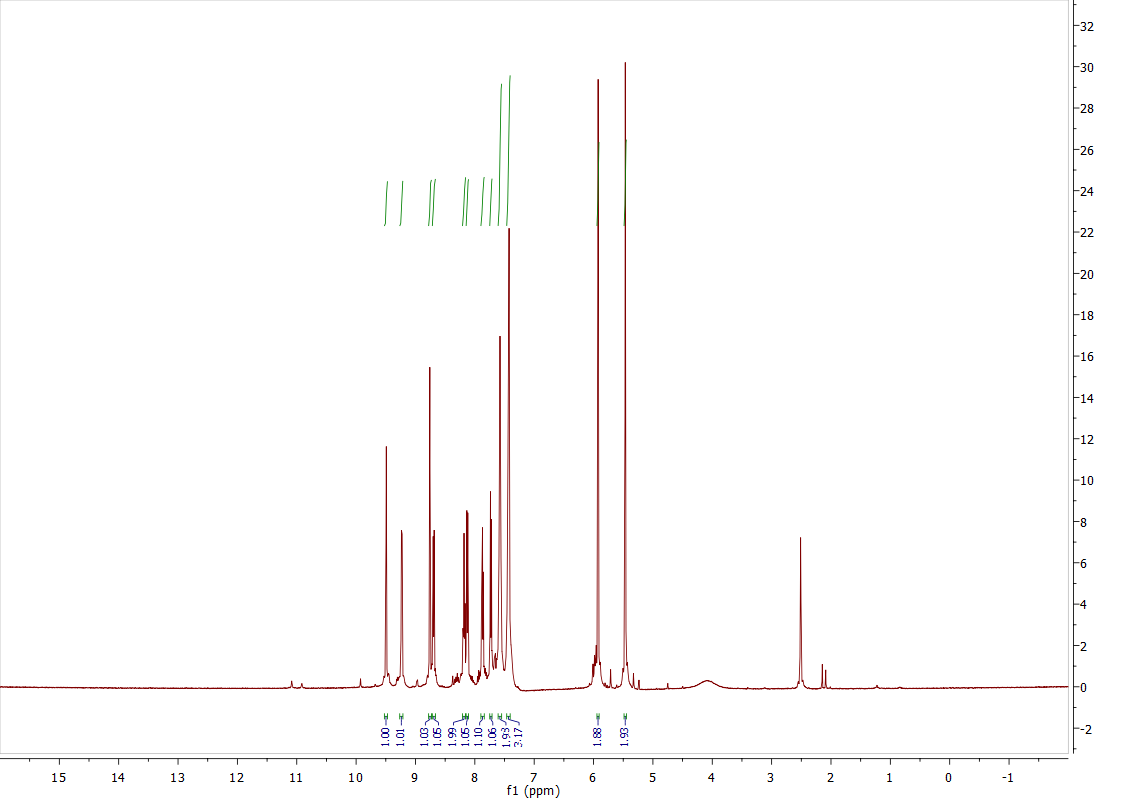
BOP-5


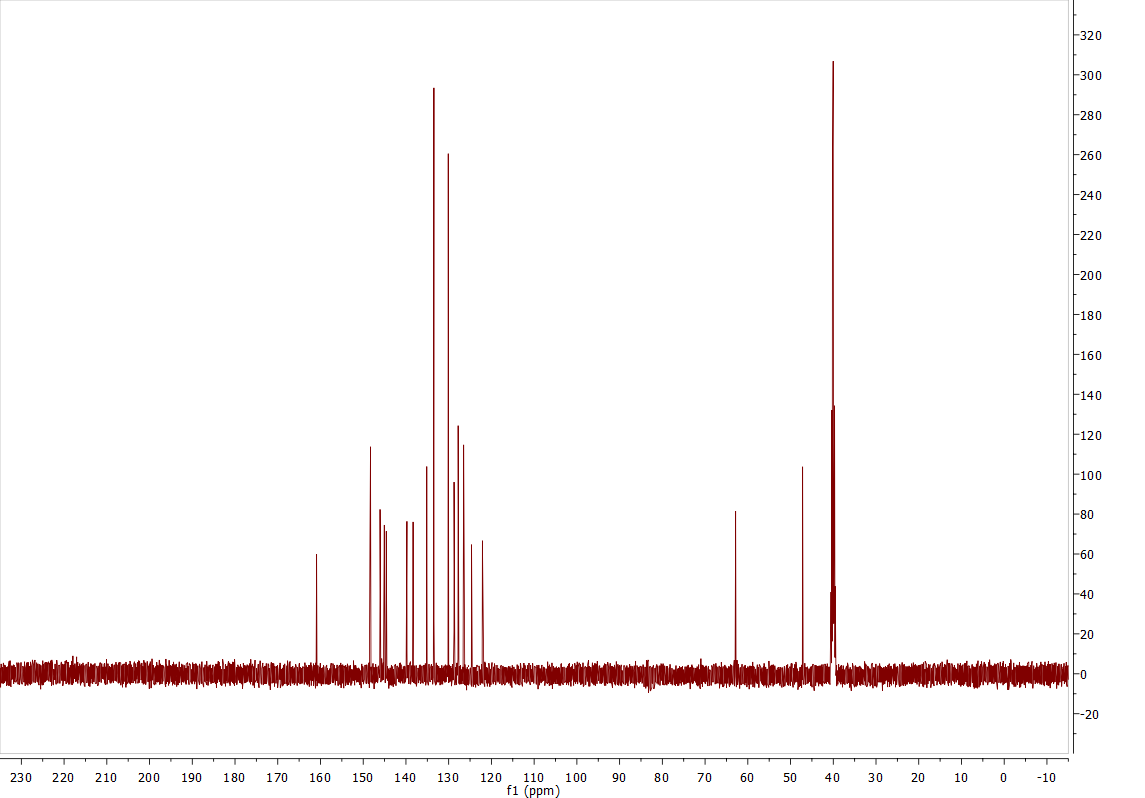


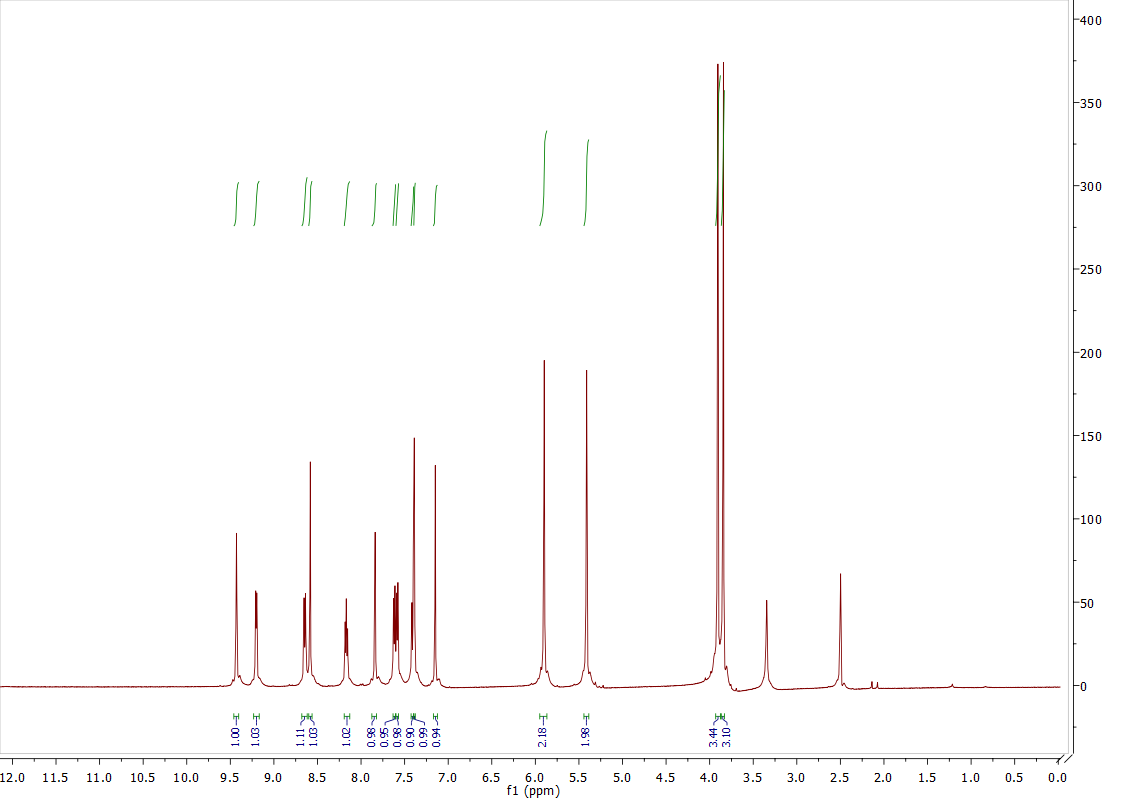
BOP-6


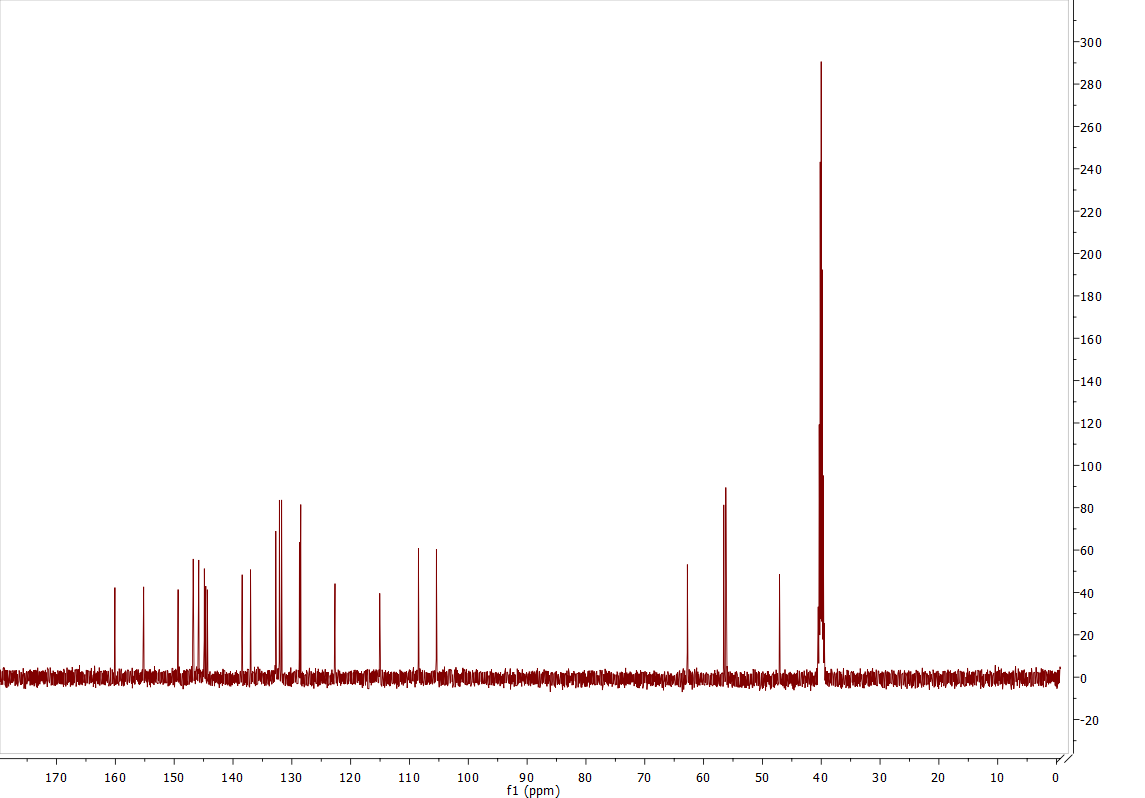


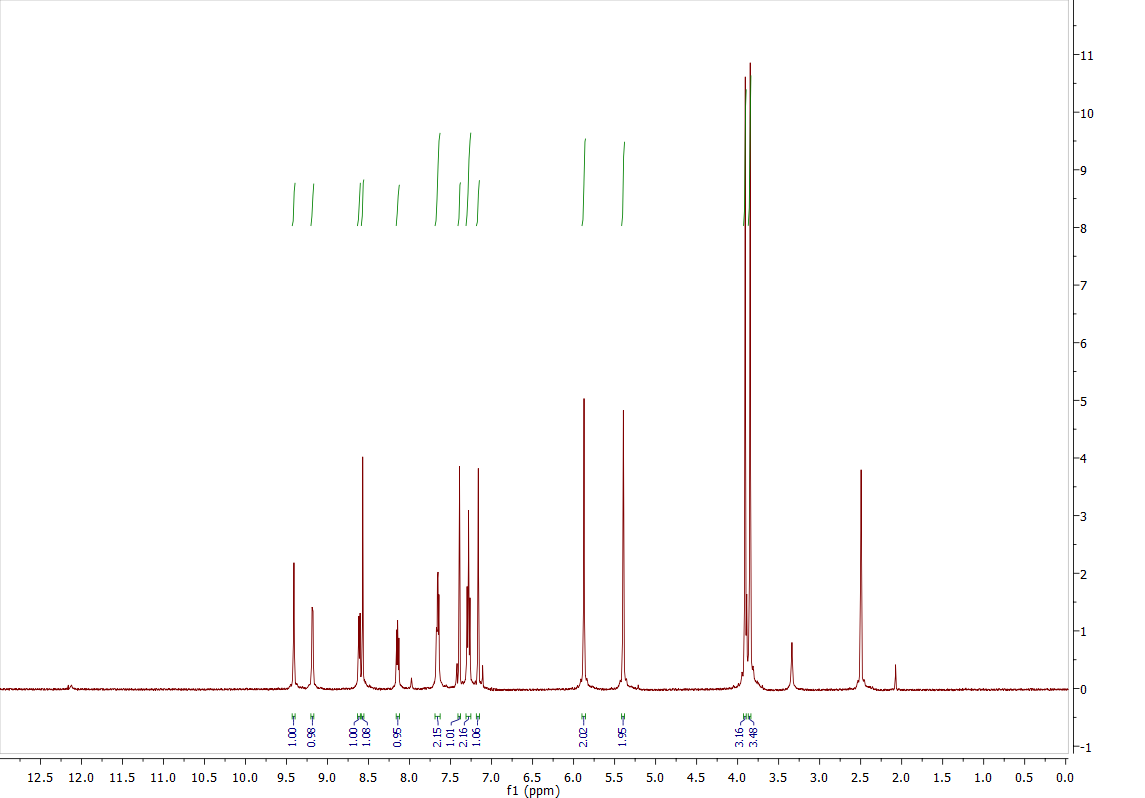
BOP_7


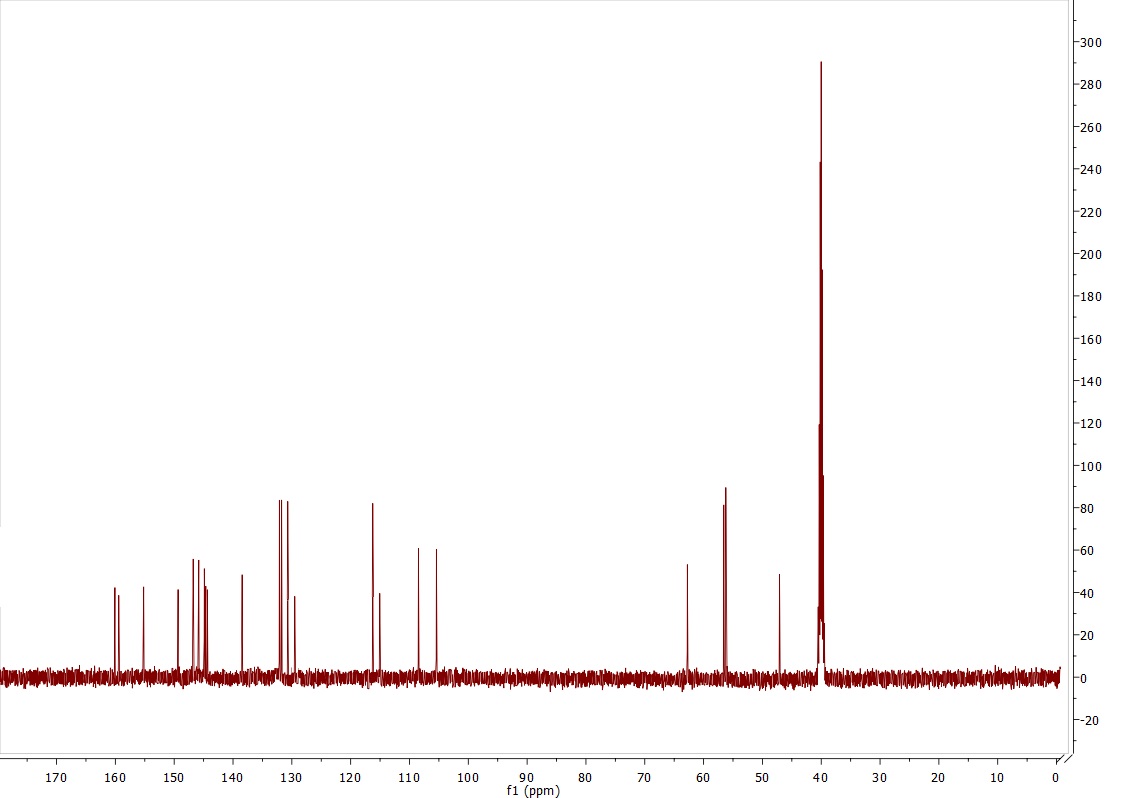


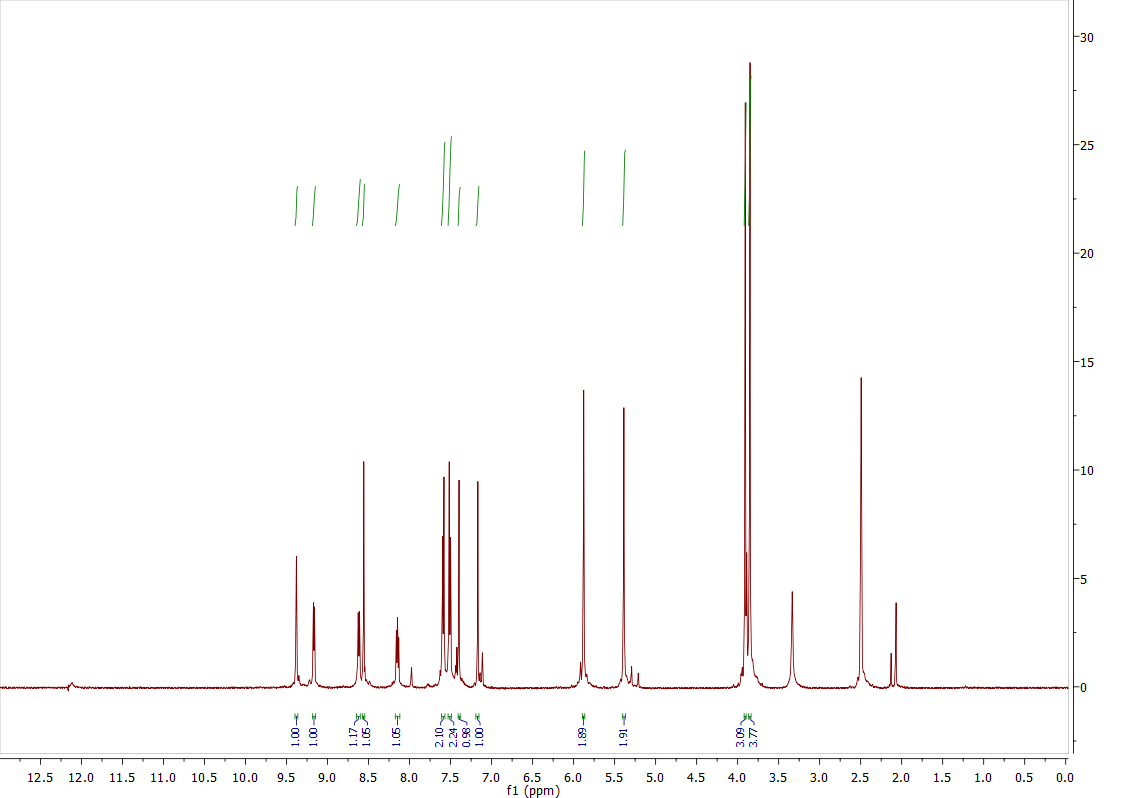
BOP-8


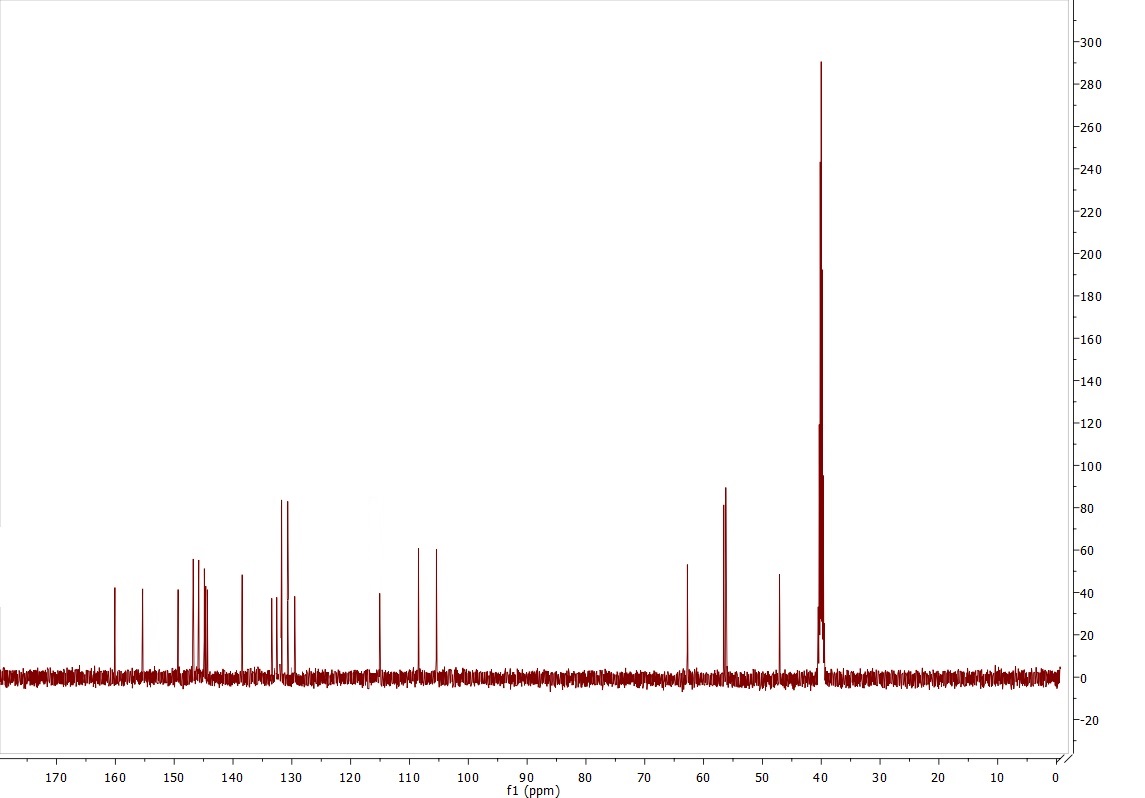


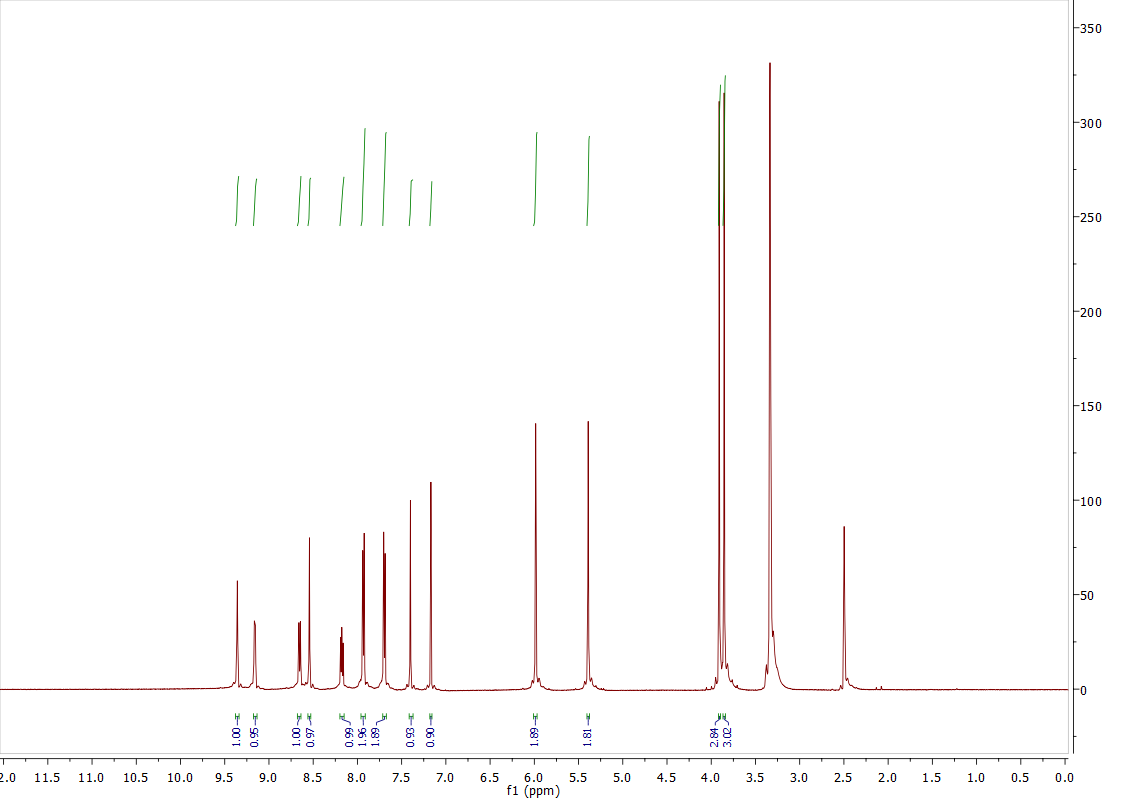
BOP-9


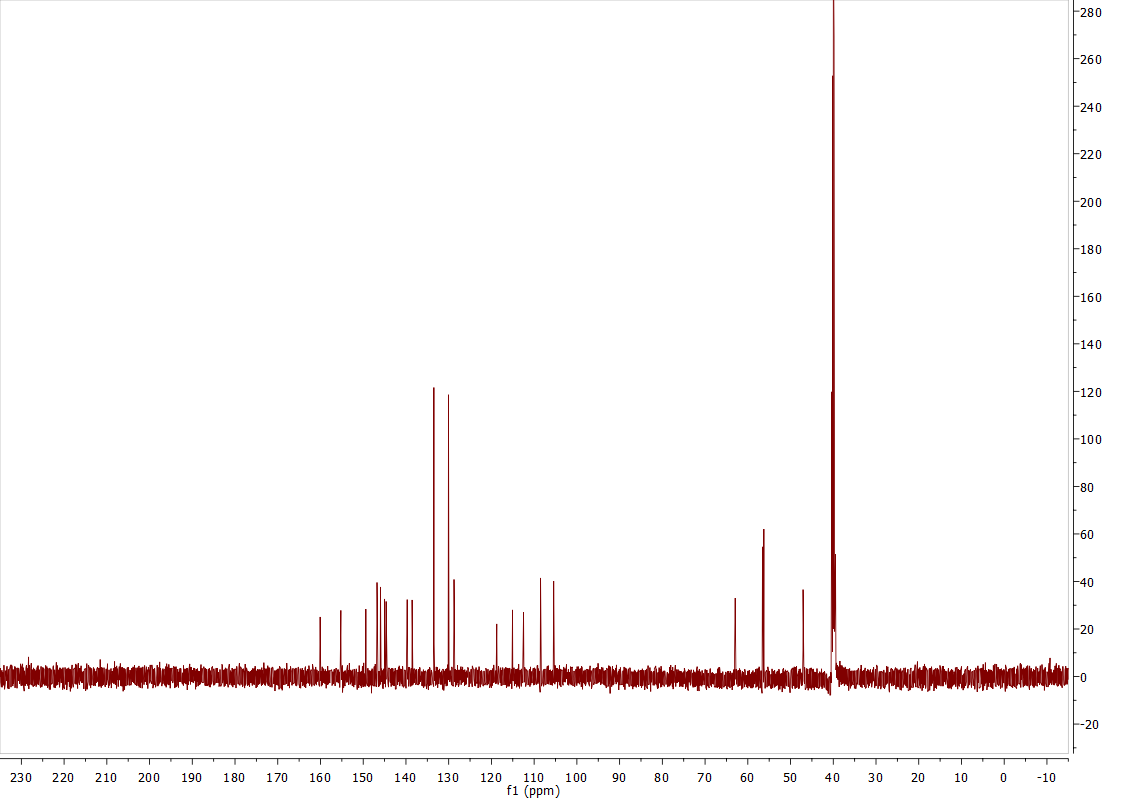


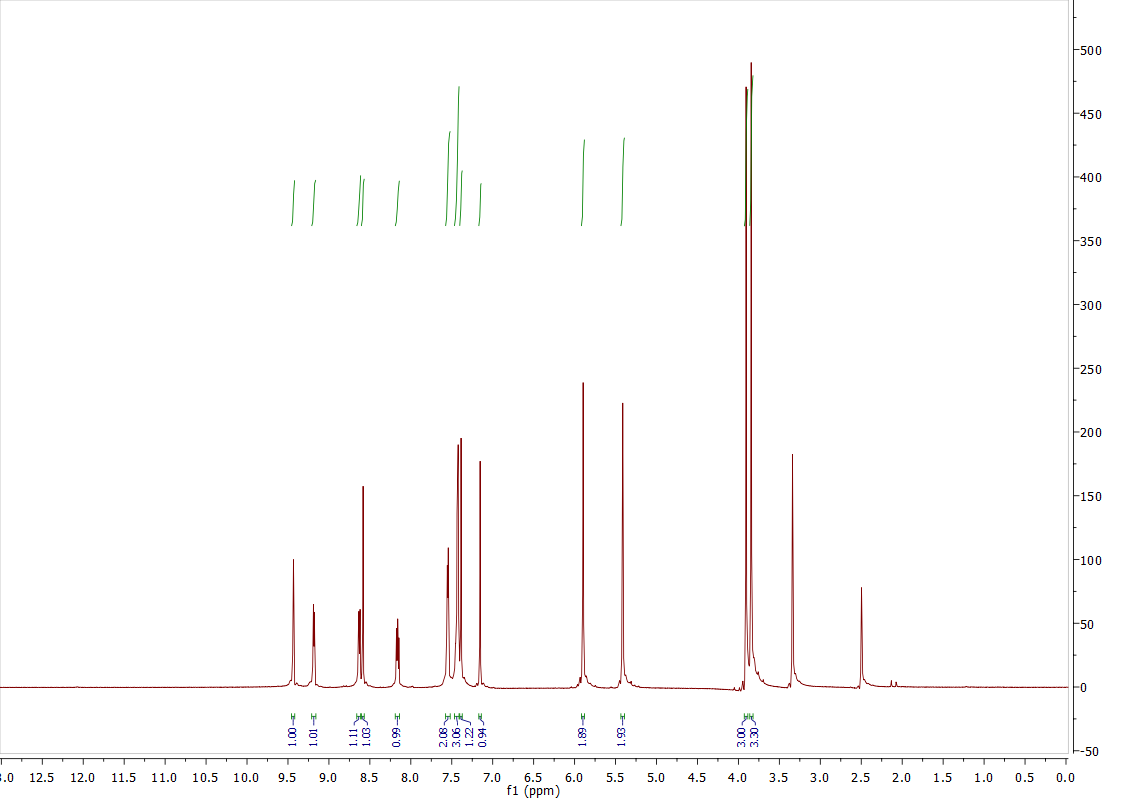
BOP-10


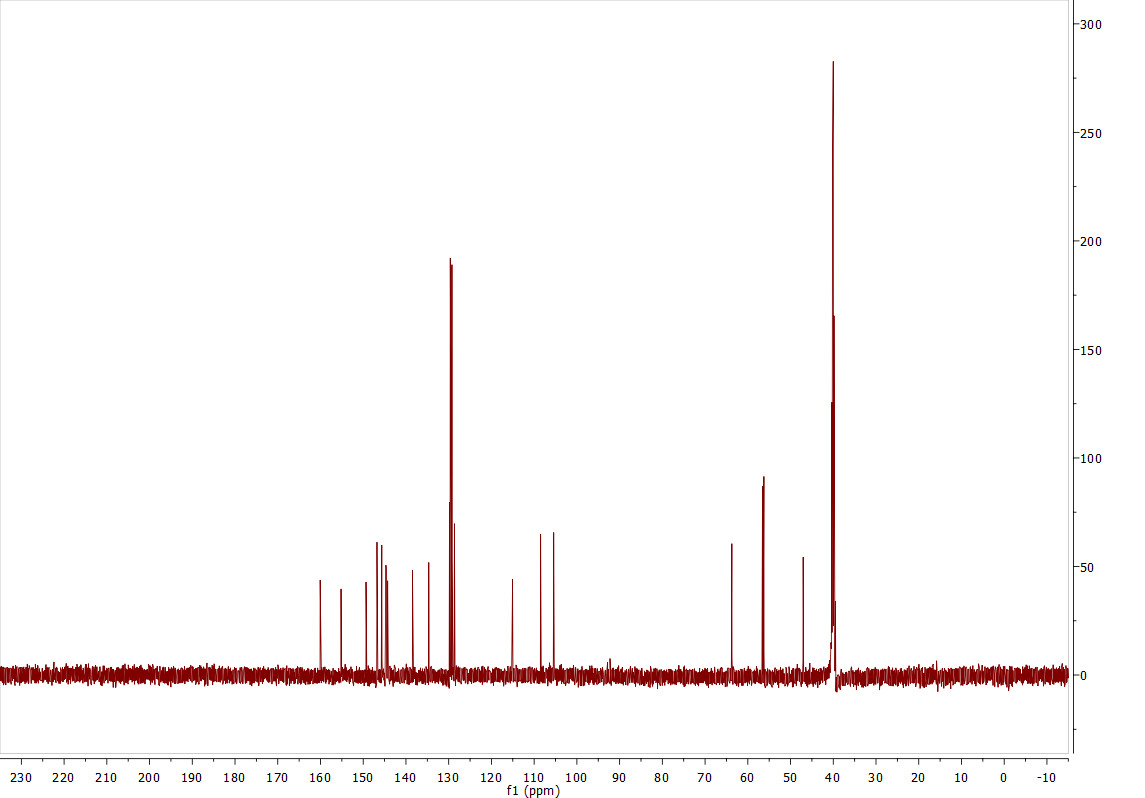

Supplement: Supplement [file mmc1.docx]
